# Supplementary material for: Singlet fission as a polarized spin generator for dynamic nuclear polarization
Source: Nat Commun. 2023 Mar 1;14:1056. doi: 10.1038/s41467-023-36698-4 (PMC9977948; doi:10.1038/s41467-023-36698-4)
Supplement: Supplementary file 2 — Supplementary Information [file 41467_2023_36698_MOESM2_ESM.pdf]

Supplementary Information for

Singlet fission as a polarized spin generator for dynamic nuclear polarization

Yusuke Kawashima, Tomoyuki Hamachi, Akio Yamauchi, Koki Nishimura, Yuma Nakashima, Saiya Fujiwara, Nobuo Kimizuka, Tomohiro Ryu, Tetsu Tamura, Masaki Saigo, Ken Onda, Shunsuke Sato, Yasuhiro Kobori, Kenichiro Tateishi, Tomohiro Uesaka, Go Watanabe, Kiyoshi Miyata, and Nobuhiro Yanai.

Correspondence to: go0325@kitasato-u.ac.jp (G.W.); kmiyata@chem.kyushu-univ.jp (K.M.) ;yanai@mail.cstm.kyushu-u.ac.jp (N.Y.)

Supplementary Note 1

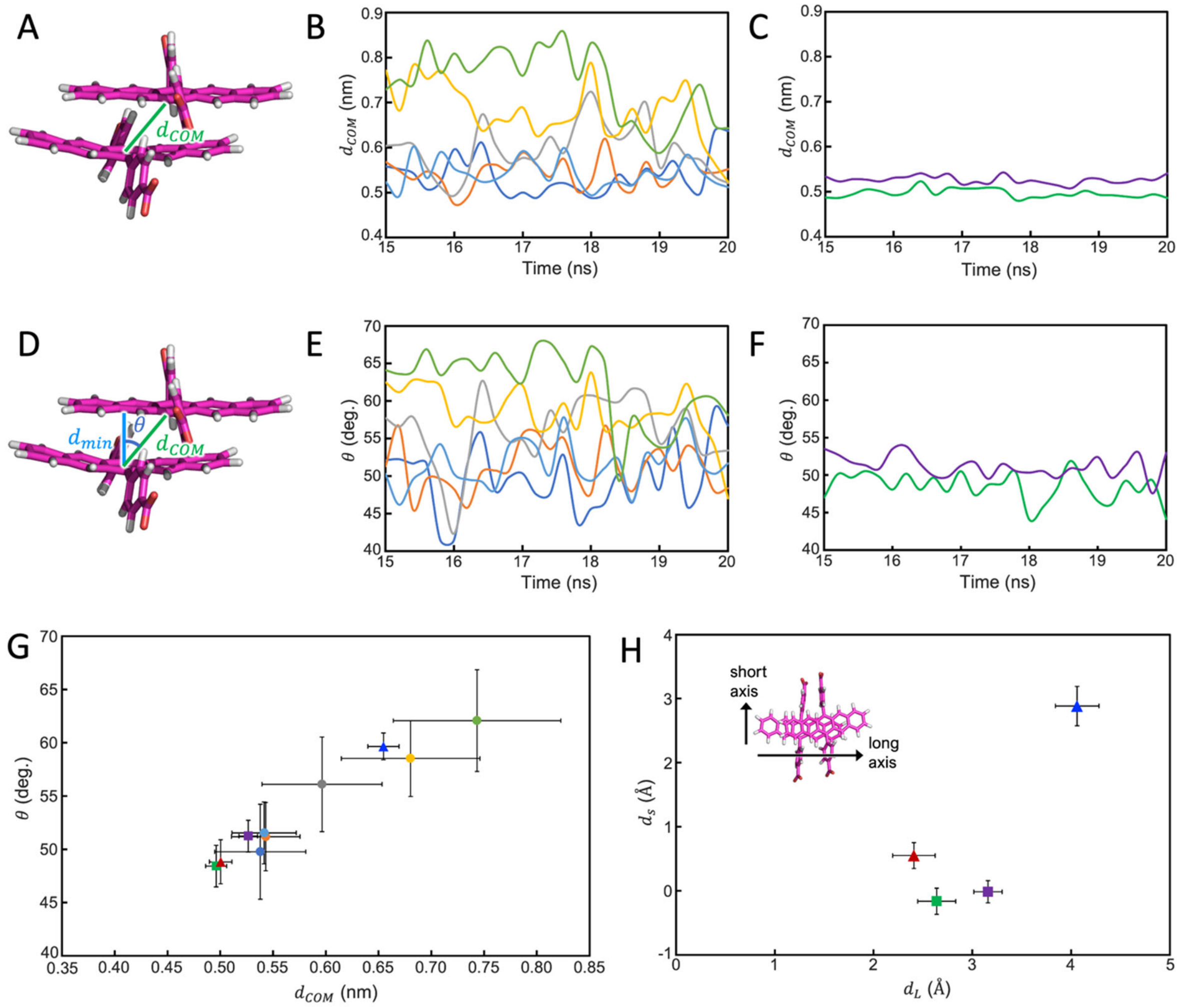

Supplementary Figure 1. (A) Definition of distance between the pentacene centers of the mass (d_{COM}) and time dependences of d_{COM} of (B) NaPDBA in water-glycerol at 300 K and (C) NaPDBA-γCD in water-glycerol at 243 K for the last 5 ns of MD simulations. (D) Definition of angle between d_{COM} and d_{min} which denotes the nearest distance between the pentacene units (θ) and time dependences of θ of (E) NaPDBA in water-glycerol at 300 K and (F) NaPDBA-γCD in water-glycerol at 243 K for the last 5 ns of MD simulations. (G) Time averaged of d_{COM} and θ: circles and squares represent those values of NaPDBA in water-glycerol at 300 K and NaPDBA-γCD in water-glycerol at 243 K, respectively; triangles represent those values of NaPDBA-γCD in water-glycerol at 243 K using initial structure in which d_{COM} is different. (H) Time averaged of d_{COM} along the direction of the short axis (d_s) and of the long axis (d_L). The curves and plots in different colors indicate different pentacene dimers in the system. The horizontal and vertical bars show the standard deviations of time series of the values during the last 5 ns of MD simulations.

.

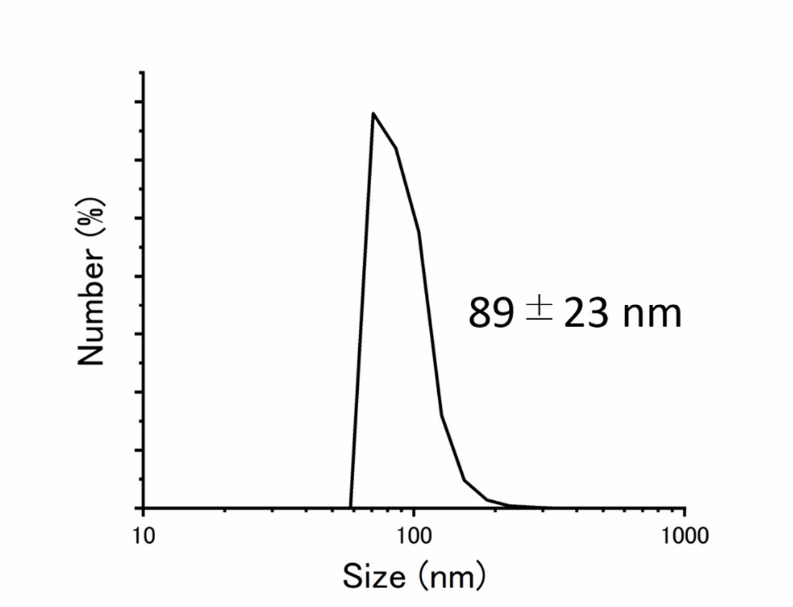

Supplementary Figure 2. DLS profile of NaPDBA in water ([NaPDBA] = 1 mM). [NaPDBA] = 1 mM dispersion was degassed by freeze pump thew cycle prior to the measurement. The data showed a particle size of 89±23 nm.

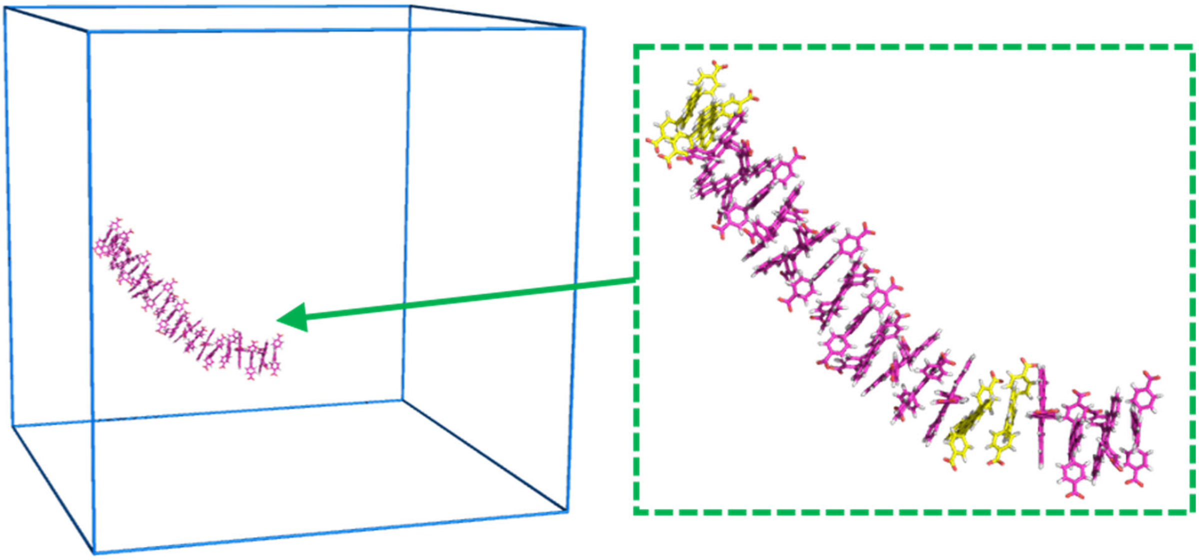

Supplementary Figure 3. MD simulation snapshots of NaPDBA in water at 300 K. Parallel oriented dimers are shown in yellow.

Evaluation of supramolecular assembly structures

We first discuss the formation of inclusion complex of NaPDBA and βCD/γCD in water-glycerol. As the concentration of βCD was increased, the absorption peak of NaPDBA slightly blue-shifted from 604 nm to 601.5 nm while passing through the isobestic point (Supplementary Figure 5), suggesting that the complexation of NaPDBA and βCD weakened the interaction between pentacene. Even in the presence of βCD, the absorption peak remained red-shifted from the that of molecularly dispersed NaPDBA in methanol (593.5 nm), suggesting that the inter-pentacene excitonic interaction was not completely eliminated probably due to the aggregation of the inclusion complex of NaPDBA and βCD. When the temperature was lowered to 143 K, the position of the absorption spectrum was red-shifted from 604 nm to 606 nm in the case of NaPDBA alone, whereas there was almost no peak shift in the case of NaPDBA-βCD (Supplementary Figure 4). Therefore, NaPDBA and βCD do indeed form an inclusion complex, and the inter-chromophore interaction is weaker than in the case of NaPDBA alone, especially at low temperatures.

On the other hand, the addition of γCD did not change the absorption spectrum of NaPDBA at room temperature with an absorption peak at 604 nm (Supplementary Figure 4). Interestingly, the absorption peak of NaPDBA was further red-shifted to 612 nm and broadened by cooling to 143 K after allowing inclusion to fully progress by standing at 243 K in the presence of γCD (Fig. 1E, S4). This suggests that the inter-pentacene excitonic interaction was enhanced by the formation of inclusion complex between NaPDBA and γCD at low temperatures.

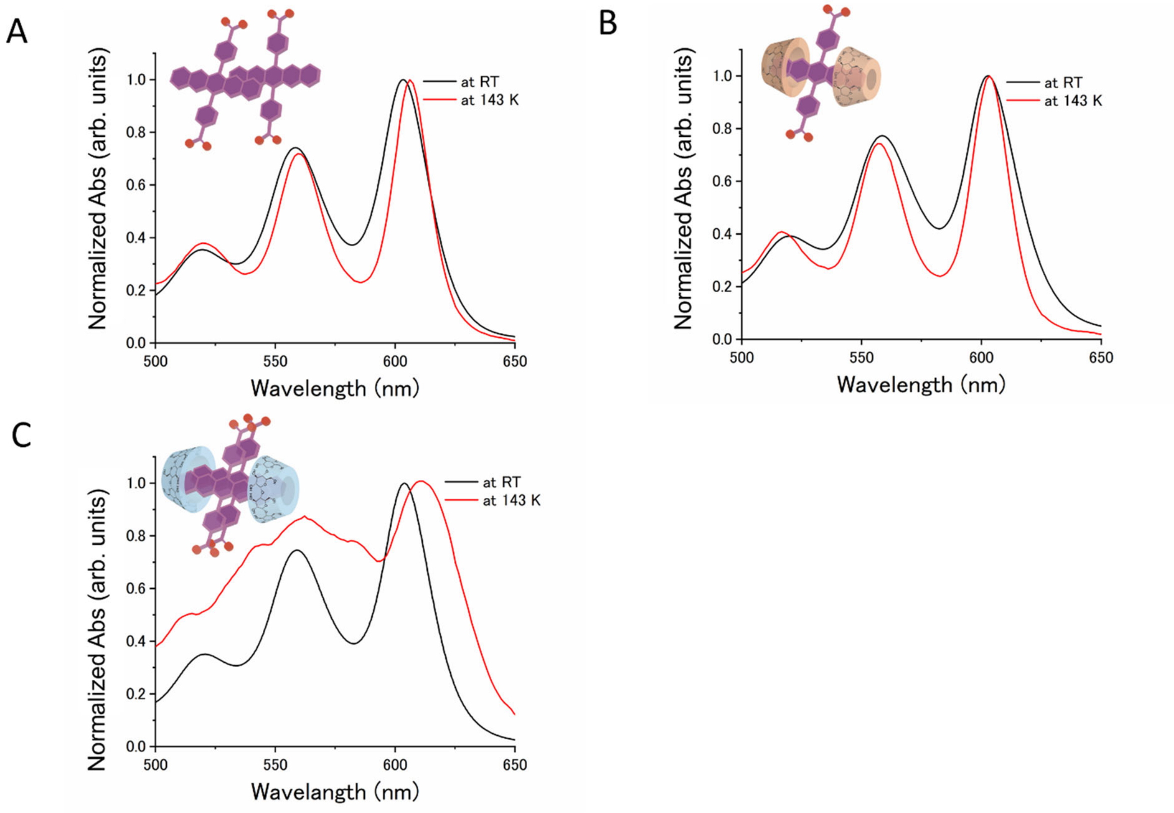

Supplementary Figure 4. Absorption spectra of (A) NaPDBA, (B) NaPDBA and βCD, and (C) NaPDBA and γCD in water-glycerol (1:1) at room temperature (black lines) and 143 K (red lines) ([NaPDBA] = 1 mM [βCD] = 0-10 mM).

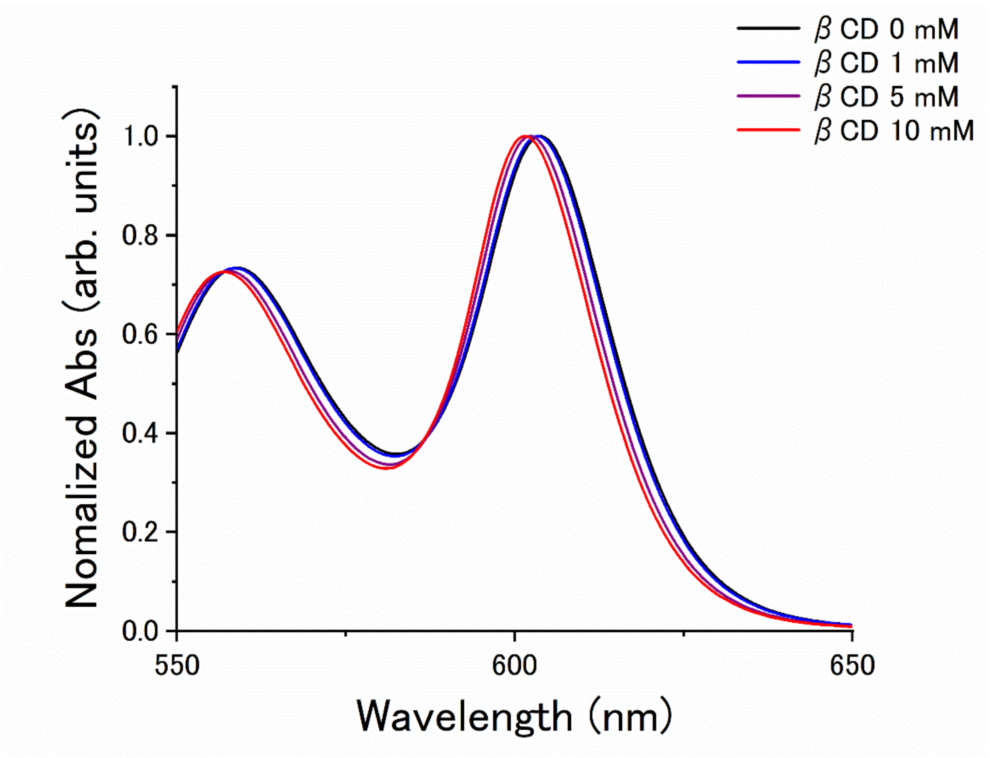

Supplementary Figure 5. Absorption spectra of NaPDBA with different concentrations of βCD in water-glycerol (1:1) at room temperature ([NaPDBA] = 1 mM [βCD] = 0-10 mM).

The complex structures between NaPDBA and βCD/γCD were further studied by NMR measurements. The NMR spectra of NaPDBA and βCD at room temperature in water-glycerol showed a change in the chemical shift of the NaPDBA-derived peak (Supplementary Figure 6A, B). Job plot by measuring the NMR spectra of NaPDBA and βCD at different concentrations suggested that the molar ratio of NaPDBA to βCD is 1:2 in their inclusion complex (Supplementary Figure 6C). This result indicates that the two βCD molecules encapsulate one NaPDBA molecule.

Due to the technical difficulty of obtaining NMR spectra with enough signal-to-noise ratio in viscous water-glycerol at low temperatures, we instead evaluated the complexation of NaPDBA with γCD in water at room temperature. The absorption spectrum of NaPDBA with γCD in water at room temperature showed a similar red-shift and broadening compared with that in water-glycerol at 143 K, and their peak positions were almost identical, indicating the formation of the similar inclusion complex (Supplementary Figure 7). Nuclear Overhauser effect spectroscopy (NOESY) NMR measurements of NaPDBA and γCD in water at room temperature revealed that there are cross peaks between the protons inside the CD ring and the pentacene skeleton of NaPDBA (Supplementary Figure 8). This result indicates that the pentacene moiety of NaPDBA is incorporated into the internal space of γCD by hydrophobic interaction. Diffusion ordered spectroscopy (DOSY) measurements indicate that the mixing of NaPDBA and γCD reduced their diffusion coefficients to similar values, which supports the complexation of NaPDBA and γCD (Supplementary Figure 9). Job plot by using NMR spectra of NaPDBA and γCD in water showed that the molar ratio of NaPDBA to γCD is 1:1 (2:2) (Supplementary Figure 10). This result suggests that two molecules of γCD encapsulate a NaPDBA dimer, which is reasonable since the inner diameter of γCD is larger than that of βCD, and strong excitonic interaction between pentacene moieties was observed in the NaPDBA-γCD inclusion complex.

The hydrophobic accommodation of NaPDBA by βCD in water at room temperature was also observed by NOESY and DOSY measurements (Supplementary Figure 8,9). The job plot in water suggested the same 1:2 molar ratio inclusion complex of NaPDBA and βCD, similar to the case in water-glycerol (Supplementary Figure 10). Absorption spectra showed that the NaPDBA-βCD inclusion complex were molecularly dispersed in water (Supplementary Figure 11), suggesting that and the NaPDBA-βCD complex aggregated in water-glycerol due to the reduced solubility of βCD.

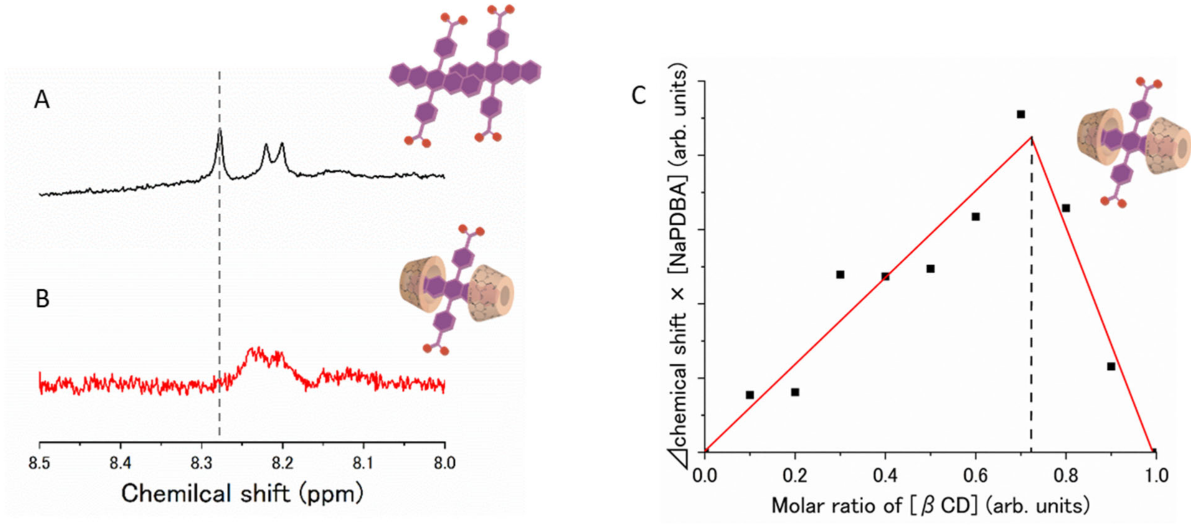

Supplementary Figure 6. ^{1}H NMR spectra of (A) NaPDBA ([NaPDBA] = 2 mM) and (B) NaPDBA and βCD ([NaPDBA] = 0.6 mM, [βCD] = 1.4 mM) in D_2O-glycerol-d_8 (1:1) at room temperature. Sodium 3-(Trimethylsilyl)-1-propanesulfonate was used as internal standard. (C) Job plot of NaPDBA and βCD in D_2O-glycerol-d_8 (1:1). The total concentration of NaPDBA and βCD was kept as 2 mM. The shift of the peak at around 8.28 ppm is shown.

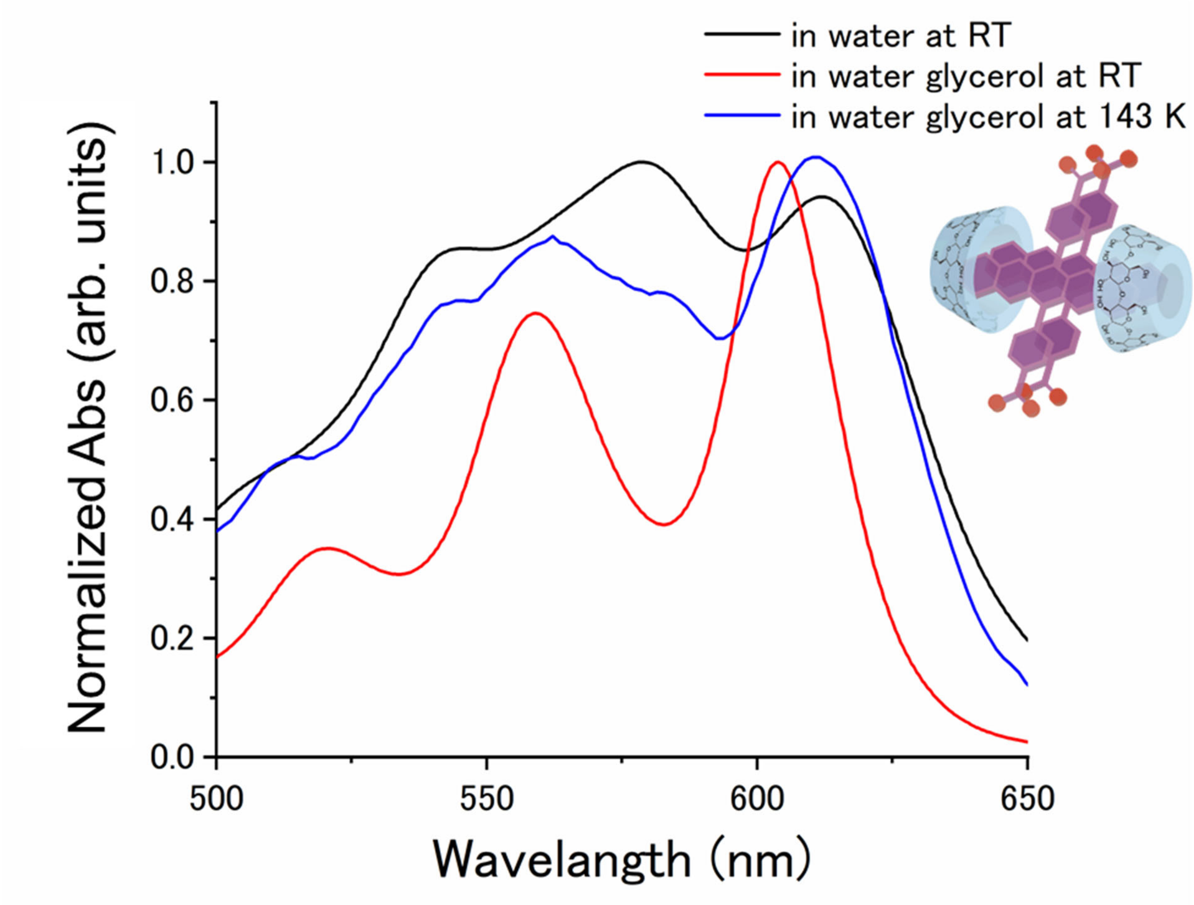

Supplementary Figure 7. Absorption spectra of NaPDBA and γCD in water at room temperature (black line), in water-glycerol (1:1) at room temperature (red line), and in water-glycerol (1:1) at 143 K (blue line) ([NaPDBA] = 1 mM, [γCD] = 5 mM).

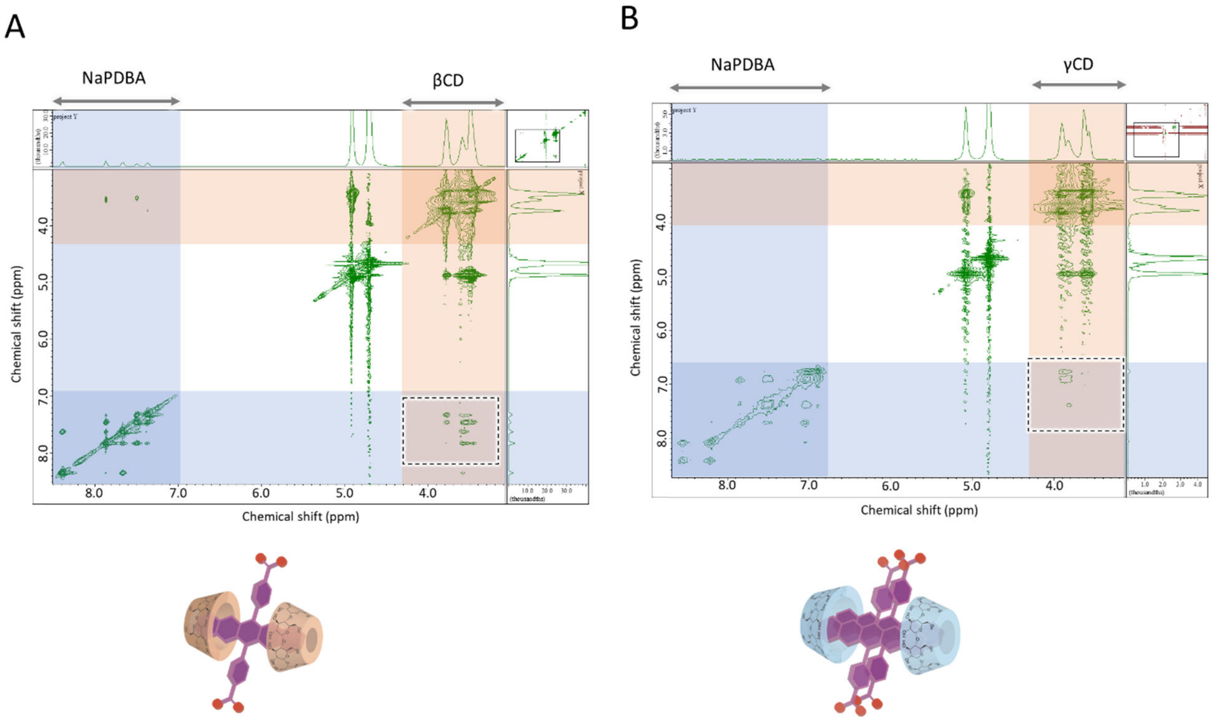

Supplementary Figure 8. ^{1}H NOESY spectra of (A) NaPDBA and βCD ([NaPDBA] = 1 mM, [βCD] = 5 mM) and (B) NaPDBA and γCD ([NaPDBA] = 1 mM, [γCD] = 5 mM) in D_2O at room temperature. Sodium 3-(Trimethylsilyl)-1-propanesulfonate was used as internal standard.

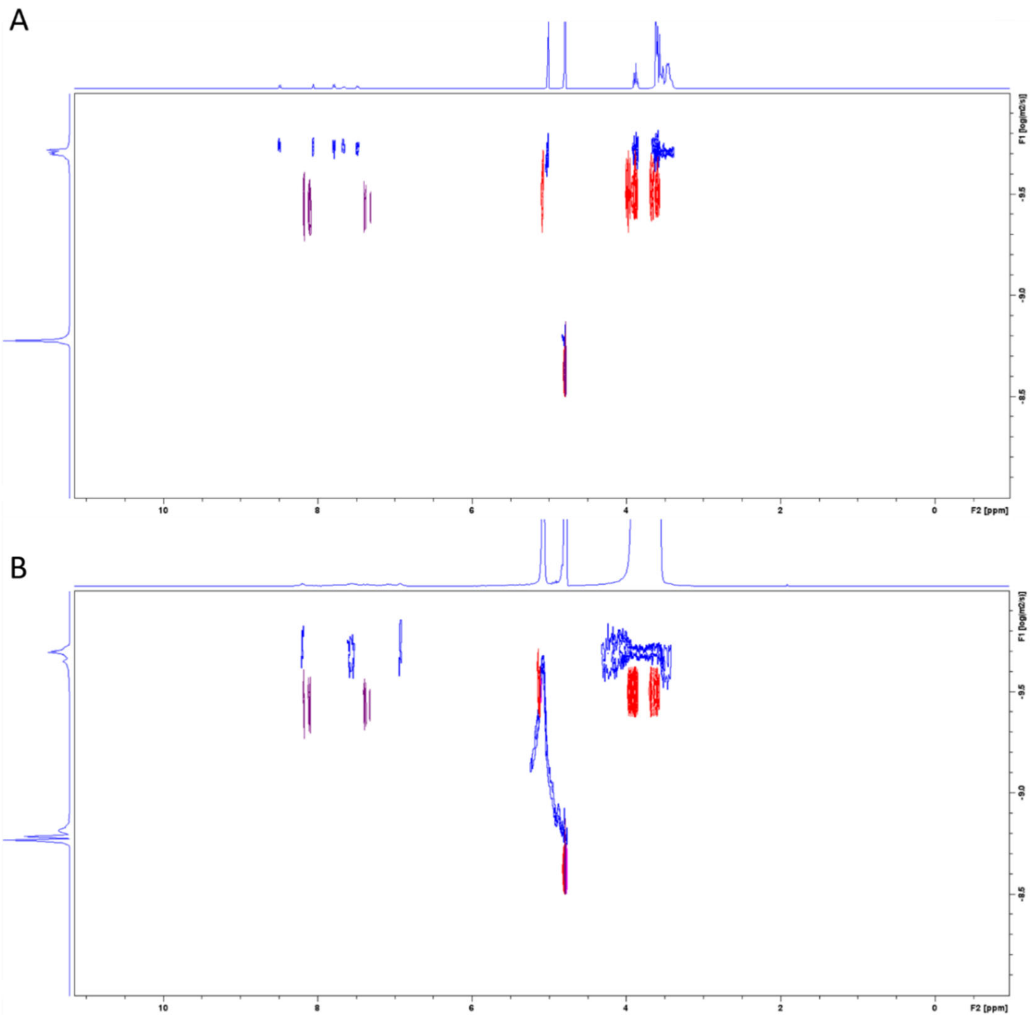

Supplementary Figure 9. DOSY spectra of NaPDBA , CD and these mixture. (A) DOSY spectra of [NaPDBA] = 1 mM (purple), [βCD] = 2 mM (red) and a mixture of [NaPDBA] = 1 mM and [βCD] = 2 mM (blue) in D_2O at room temperature. Residual water was used as internal standard. (B) DOSY spectra of [NaPDBA] = 1 mM (purple), [γCD] = 2 mM (red) and a mixture of [NaPDBA] = 1 mM and [γCD] = 2 mM (blue) in D2O at room temperature. Residual water was used as internal standard.

Deuterated water was used as the solvent because the viscosity of the water-glycerol mixture was too high for reliable DOSY measurements. γCD-derived NMR peaks were observed around 3.5-5.0 ppm and NaPDBA-derived NMR peaks around 7.5-8.5 ppm, both showing a decrease in diffusion coefficient by the mixing of γCD and NaPDBA. The diffusion coefficient of NaPDBA decreased from 2.7×10^{-10} m^2 s^{-1} to 1.6×10^{-10} m^2 s^{-1} and that of γCD decreased from 2.2×10^{-10} m^2 s^{-1} to 2.0×10^{-10} m^2 s^{-1} upon mixing. Importantly, the diffusion coefficients of NaPDBA and γCD were almost the same after mixing, which supports that NaPDBA and γCD form a complex. Similarly, the mixing of NaPDBA and βCD resulted in the decrease of the diffusion coefficients of NaPDBA and βCD from 2.7×10^{-10} m^2 s^{-1} to 1.8×10^{-10} m^2 s^{-1} and from 2.3×10^{-10} m^2 s^{-1} to 2.0×10^{-10} m^2 s^{-1}, respectively, confirming the formation of the NaPDBA-βCD complex.

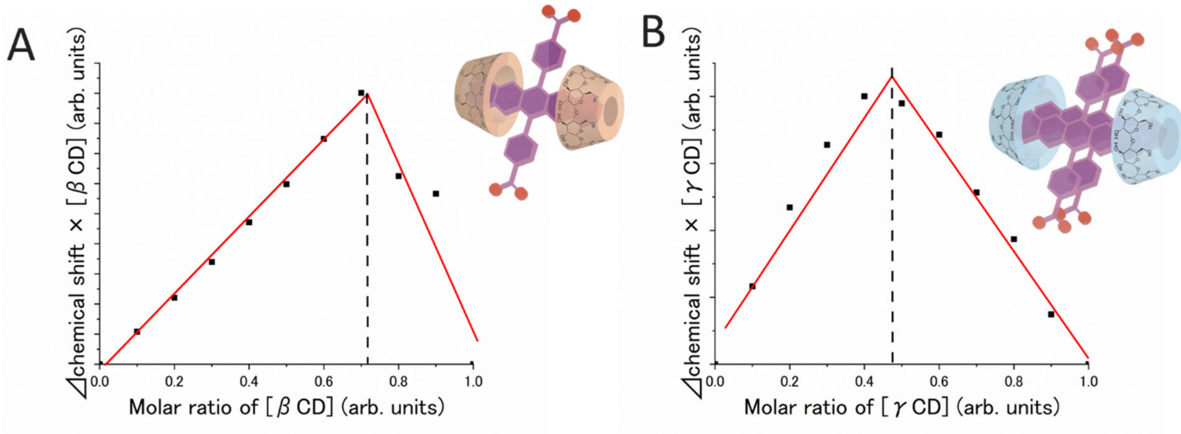

Supplementary Figure 10. (A) Job plot of NaPDBA and βCD in D_2O. The total concentration of NaPDBA and βCD was kept as 2 mM. The shift of the peak at around 3.78 ppm is shown. (B) Job plot of NaPDBA and γCD in D_2O. The total concentration of NaPDBA and γCD was kept as 1 mM. The shift of the peak at around 3.93 ppm is shown. Sodium 3-(Trimethylsilyl)-1-propanesulfonate was used as internal standard.

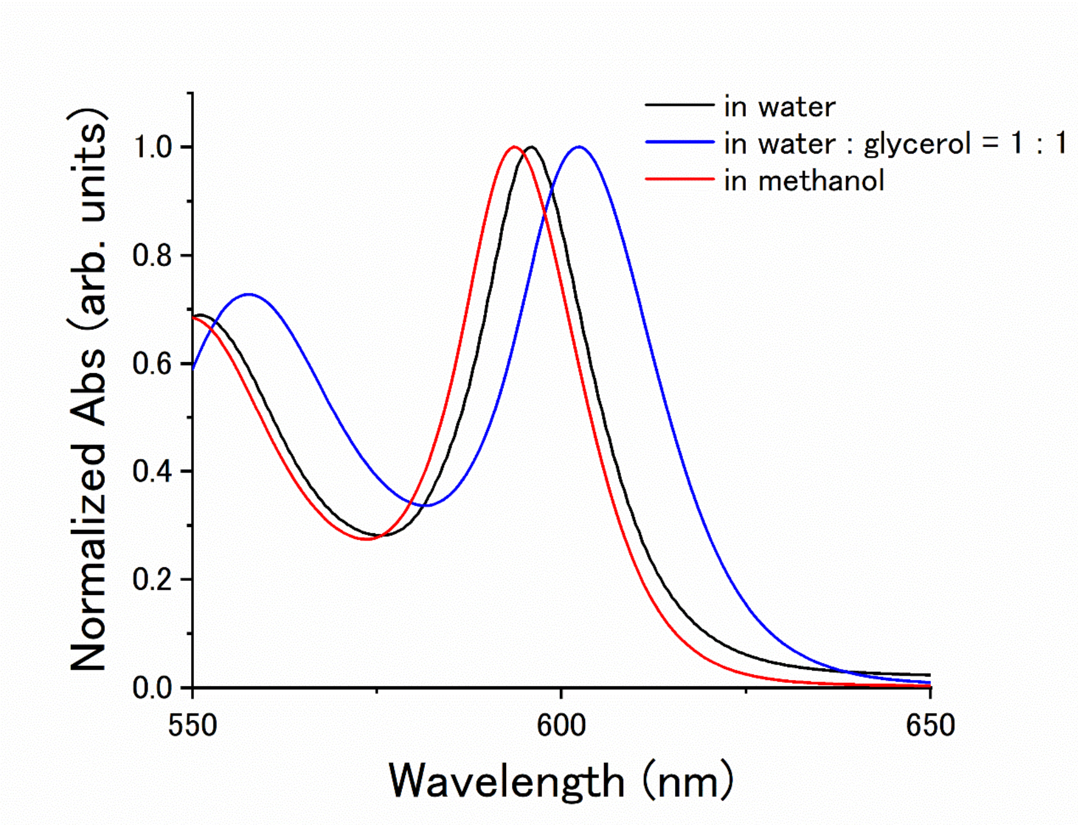

Supplementary Figure 11. Absorption spectra of NaPDBA and βCD in water (black), in water-glycerol (1:1) (blue) and only NaPDBA in methanol (red) at room temperature ([NaPDBA] = 1 mM, [βCD] = 5 mM).

In the MD simulation, the 1:2 inclusion complex of NaPDBA-βCD was found to be stable in water-glycerol at room temperature (Supplementary Figure 13). When the simulation was started from the initial aggregated structure of the inclusion complexes, no dispersion behavior was observed, supporting that the excitonic interaction between pentacenes observed in the absorption spectra is caused by the aggregation of the inclusion complexes.

The PMFs for pulling away one γCD molecule of the 2:2 inclusion complex of NaPDBA-γCD in water-glycerol until it unfolded the NaPDBA dimer were 7.9 ± 2.1 and 21.1 ± 2.4 kJ/mol. Since the thermal energy of the complex consisting of 4 molecules at 300 K can be estimated about 10 kJ/mol, the complex of NaPDBA-γCD in water-glycerol at 300 K should not be energetically stable. It indicates that some NaPDBA molecules might be dispersed in the system without forming the complex.

Both 1:2 inclusion complex of NaPDBA-βCD and 1:1 complex of NaPDBA-γCD were found to be stable and dispersed in water at room temperature in the MD simulations (Supplementary Figure 14).

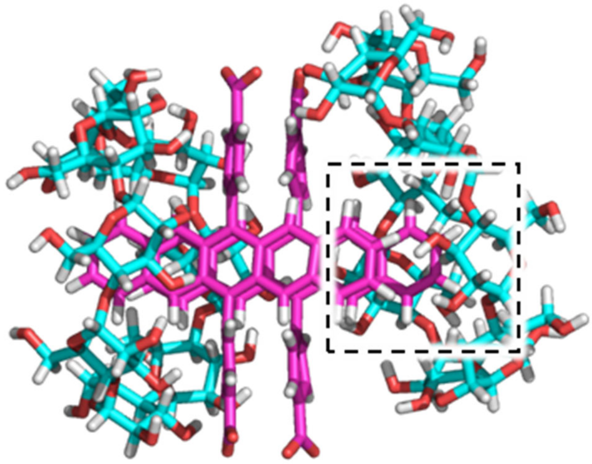

Supplementary Figure 12. MD simulation snapshots of NaPDBA and γCD ([NaPDBA] = 1 mM, [γCD] = 5 mM) in water-glycerol (1:1) at 300 K. The dotted area shows that NaPDBA and γCD are about to be detached.

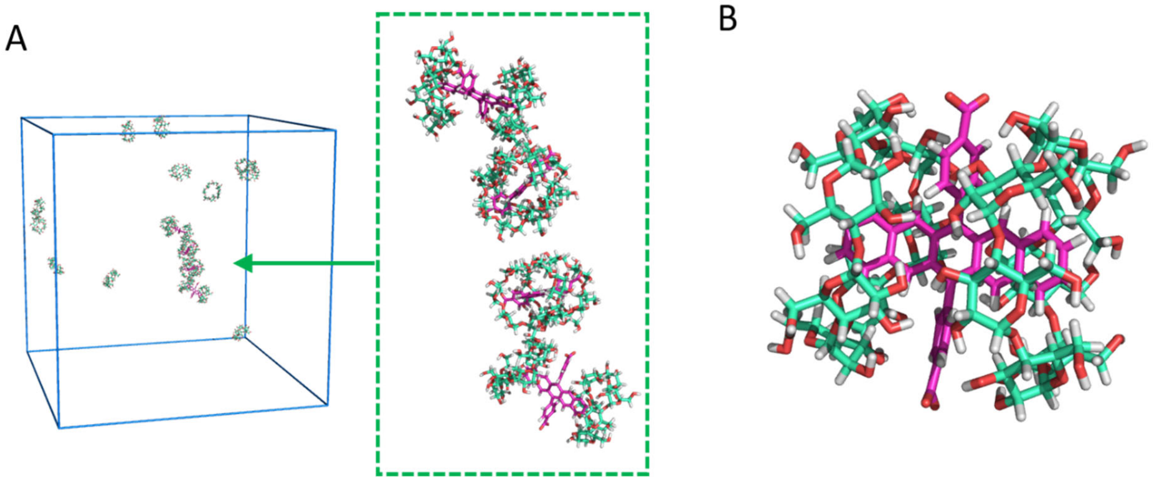

Supplementary Figure 13. MD simulation snapshots of (A) NaPDBA and βCD ([NaPDBA] = 1 mM, [βCD] = 5 mM) in water-glycerol (1:1) at 300 K. (B) One of the snapshots of NaPDBA-βCD inclusion complexes.

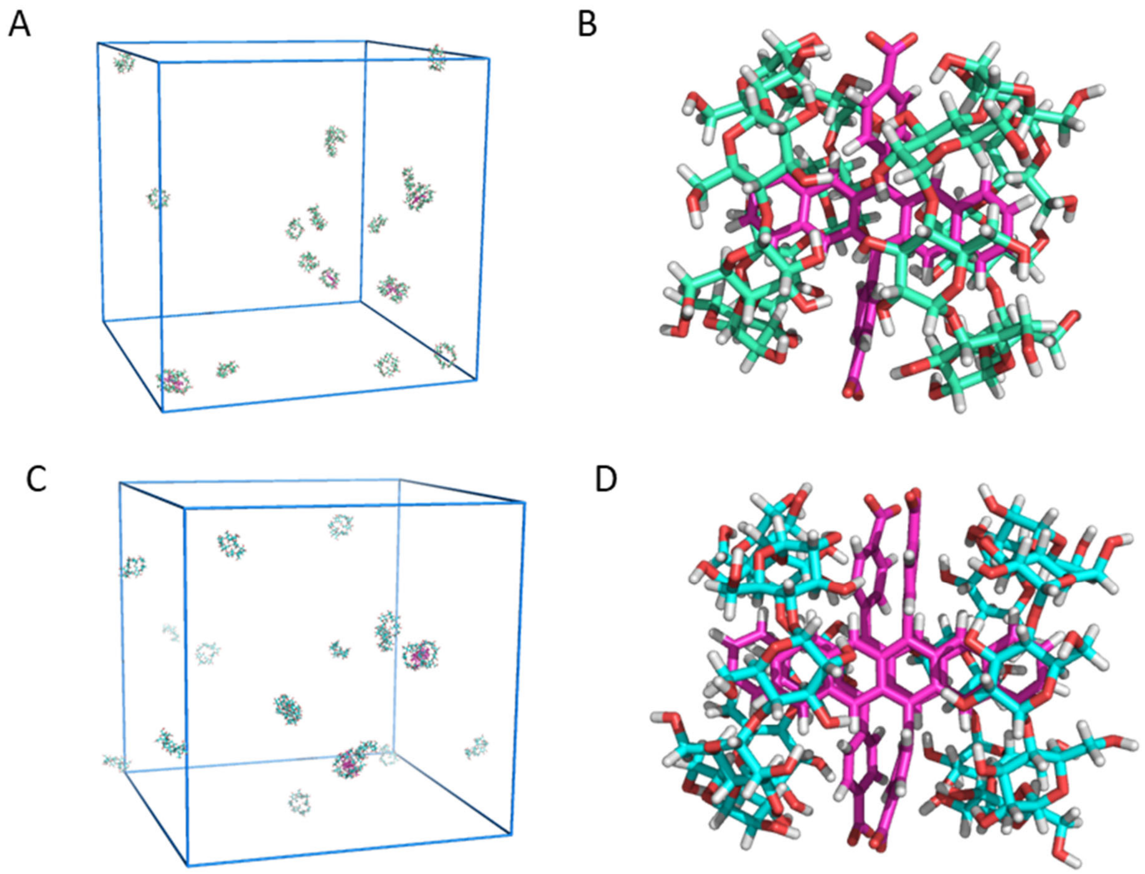

Supplementary Figure 14. (A, B) MD simulation snapshots of NaPDBA and βCD ([NaPDBA] = 1 mM, [βCD] = 5 mM) in water at 300 K. (C, D) MD simulation snapshots of NaPDBA and γCD ([NaPDBA] = 1 mM, [γCD] = 5 mM) in water at 300 K.

.

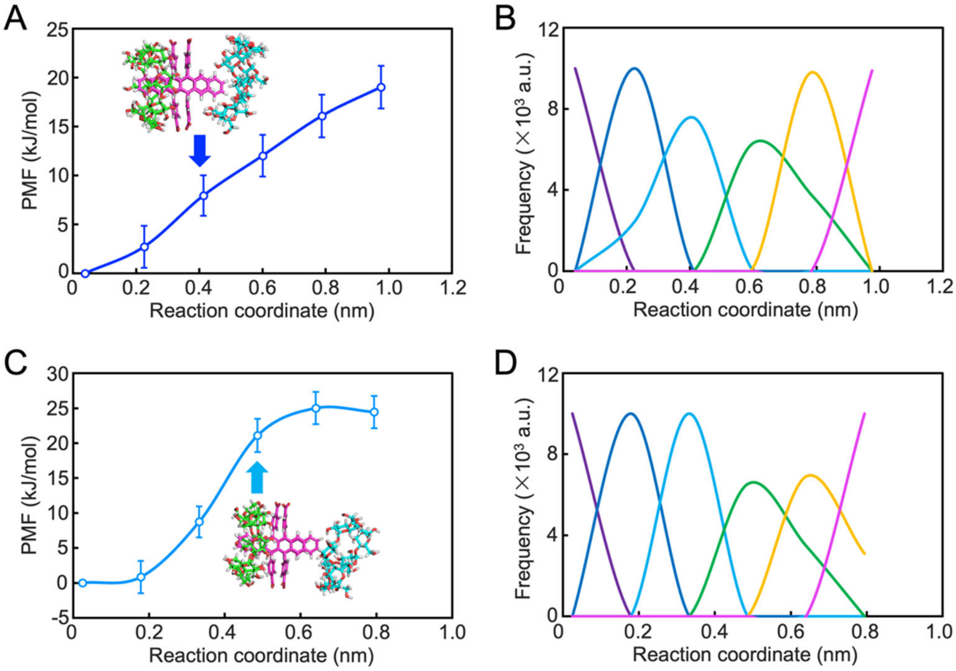

Supplementary Figure 15. PMF profiles of 2:2 inclusion complex of NaPDBA-γCD in water-glycerol at 300 K (A) and at 243 K (C). Each inset snapshot illustrates the top view of the NaPDBA-γCD complex at the moment of its collapse. Apparent probability density of 2:2 inclusion complex of NaPDBA-γCD in water-glycerol at 300 K (B) and at 243 K (D). The curves drawn in different colors were calculated from each US simulation. The error bars depict only the statistical uncertainty from the connection of probability density by WHAM.

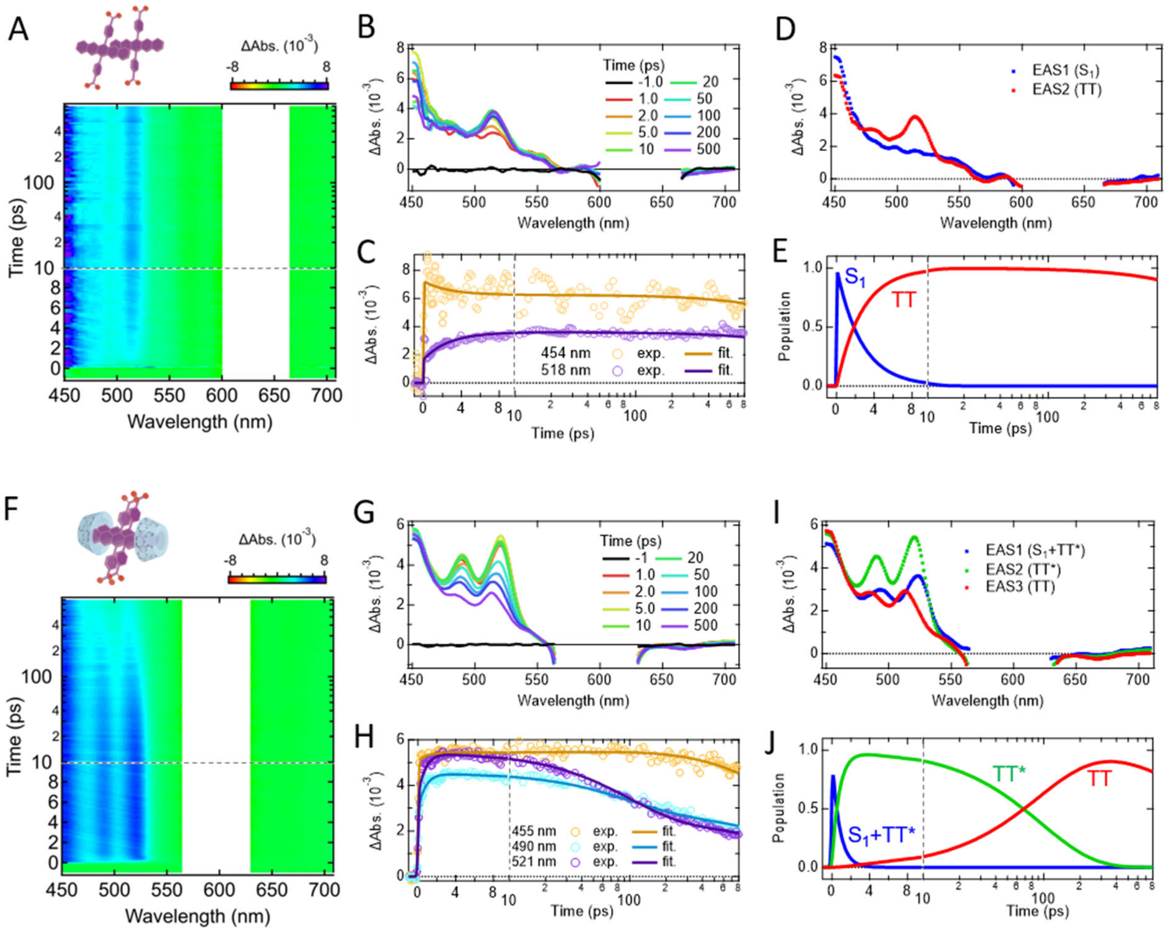

Supplementary Figure 16. fs-TAS measurements of the supramolecular assemblies. (This figure displays the entire wavelength range of Fig. 3) Overview of fs-TAS analysis of (A-E) NaPDBA and (F-J) NaPDBA-γCD in water-glycerol (1:1) at 143 K ([NaPDBA] = 1 mM, [γCD] = 5 mM). (A, F) Pseudo-2D plots of experimentally observed fs-TAS (excitation: 635 nm for NaPDBA and 600 nm for NaPDBA-γCD), (B, G) spectral evolution of the TAS, and (C, H) temporal change of transient absorption at selected wavelengths and fitting curves from global analysis. (D, I) Evolution-associated spectra and (E, J) corresponding concentration kinetics obtained from global analysis based on sequential models.

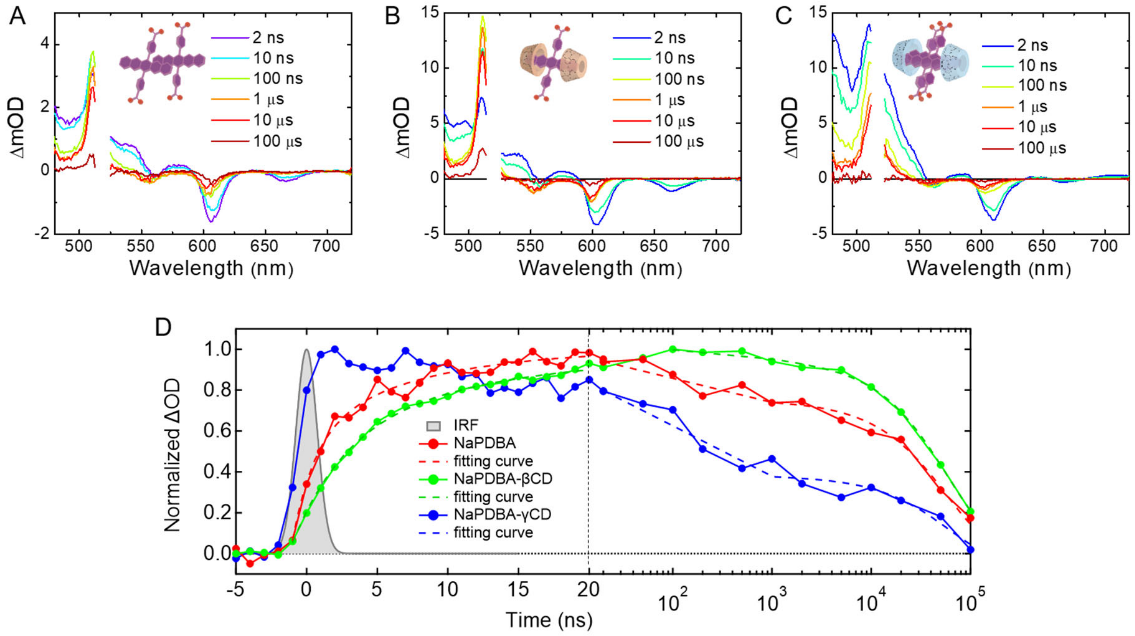

Supplementary Figure 17. Overview of ns-TAS spectral evolution of (A) NaPDBA, (B) NaPDBA-βCD, and (C) NaPDBA-γCD (excitation: 520 nm). (D)Kinetic traces of the transient absorption at 510 nm from -10 ns to 20 μs. The time constants of the rise components of T_1-T_n absorption observed at 510 nm were 3.4±1.9 and 23±2.4 ns for NaPDBA, 5.6±0.6 and 38±5.0 ns for NaPDBA-βCD, 2.6±1.7 ns for NaPDBA-γCD. The time constants of the decay components of T_1-T_n absorption observed at 510 nm were 0.081±0.062, 2.4±3.4 and 73±2.8 μs for NaPDBA, 0.585±0.558 and 58±3.7 μs for NaPDBA-βCD, 0.010±0.007, 0.222±0.082 and 62±26 μs for NaPDBA-γCD. We confirmed that the population lifetimes were long enough to expect DNP.

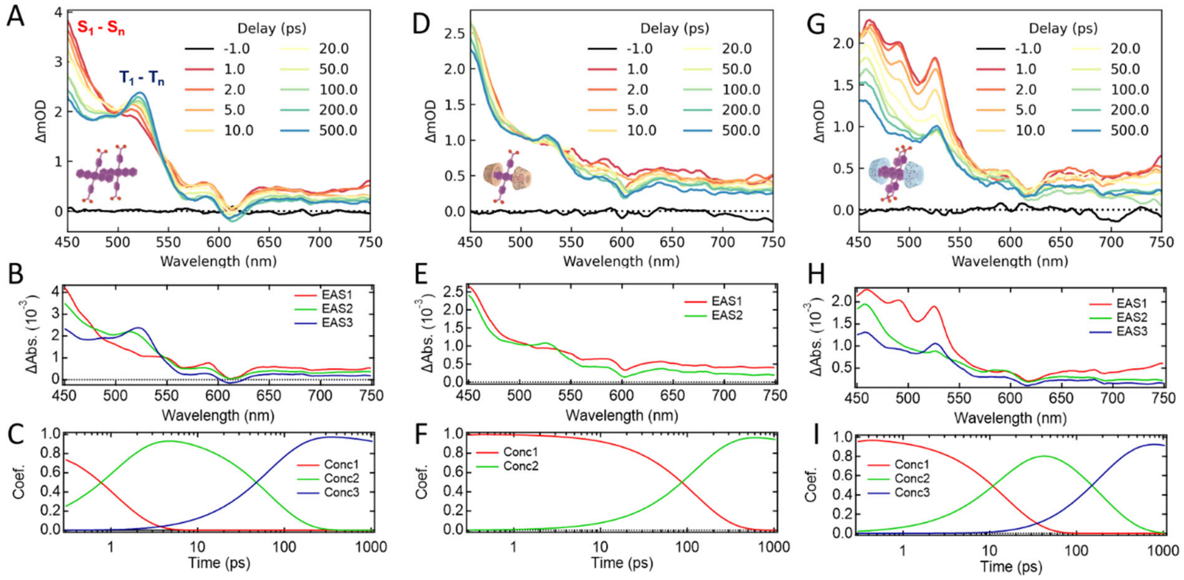

Supplementary Figure 18. Overview of fs-TAS analysis of (A-C) NaPDBA water solution at room temperature, (D-F) NaPDBA water solution with βCD, and (G-H) NaPDBA water solution with γCD. (A, D, G) Experimentally observed fs-TAS (excitation: 400 nm). (B, E, H) Evolution associated spectra and (C, F, I) respective concentration kinetics obtained from target analysis based on sequential models. While NaPDBA water solution and one with γCD showed distinct T_1-T_n transition at 520 nm observed in a few picoseconds owing to SF, one with βCD showed negligible SF indicated by the almost S_1-like spectral feature of EAS2.

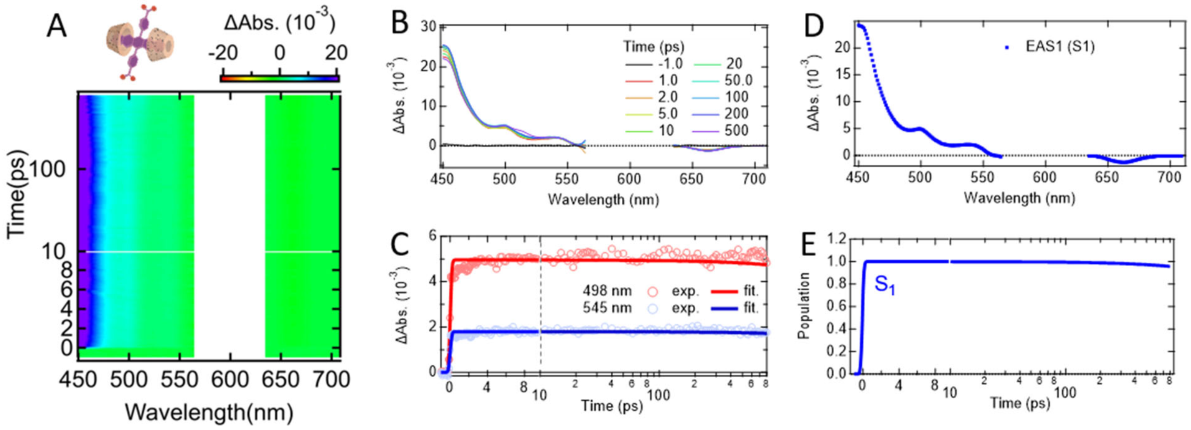

Supplementary Figure 19. Overview of fs-TAS analysis of NaPDBA-βCD in water-glycerol (1:1) at 143 K ([NaPDBA] = 1 mM, [βCD] = 5 mM). (A) Pseudo-2D plots of experimentally observed fs-TAS (excitation: 600 nm), (B) spectral evolution of the TAS, and (C) temporal change of transient absorption at selected wavelength and fitting curve resulted from global analysis. (D) Evolution associated spectra and (E) corresponding concentration kinetics obtained from global analysis with one transient.

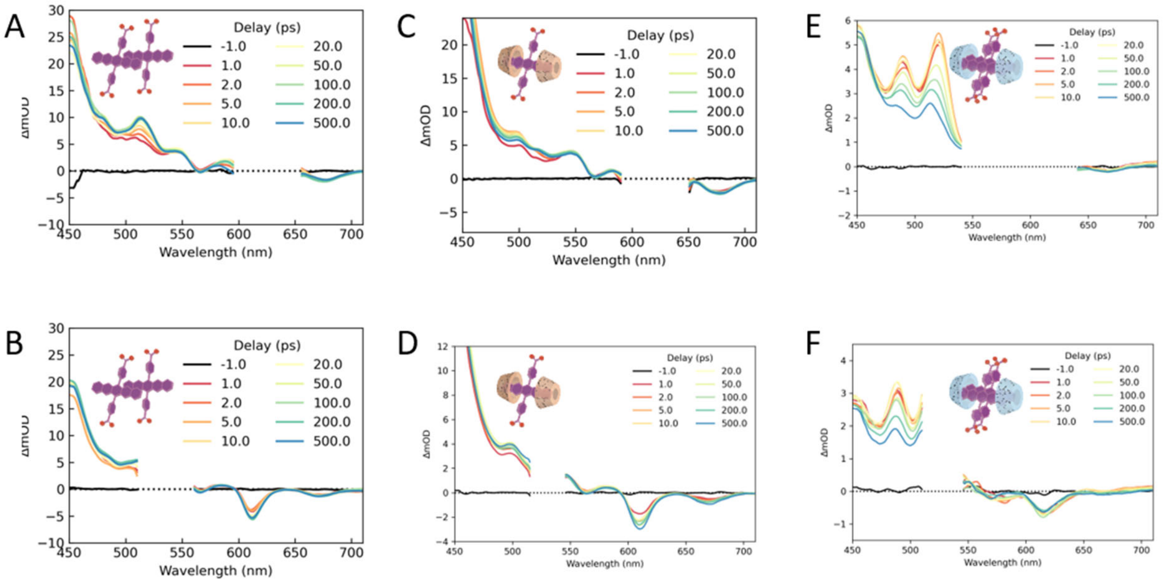

Supplementary Figure 20. Excitation wavelength dependence of fs-TAS. Low-temperature fsTAS (143 K) analysis of the NaPDBA water-glycerol solution. ([NaPDBA] = 1 mM). (A, B) fs-TAS of NaPDBA solution excited with (A) 620 nm, and (B) 527 nm; (C, D) fs TAS of NaPDBA-βCD excited with (C) 625 nm, and (D) 527 nm; (E, F) fs-TAS of NaPDBA-γCD excited with (E) 600 nm, and (F) 527 nm. Because of strong scattering from pump pulses, TA data around the pump wavelength were not available.

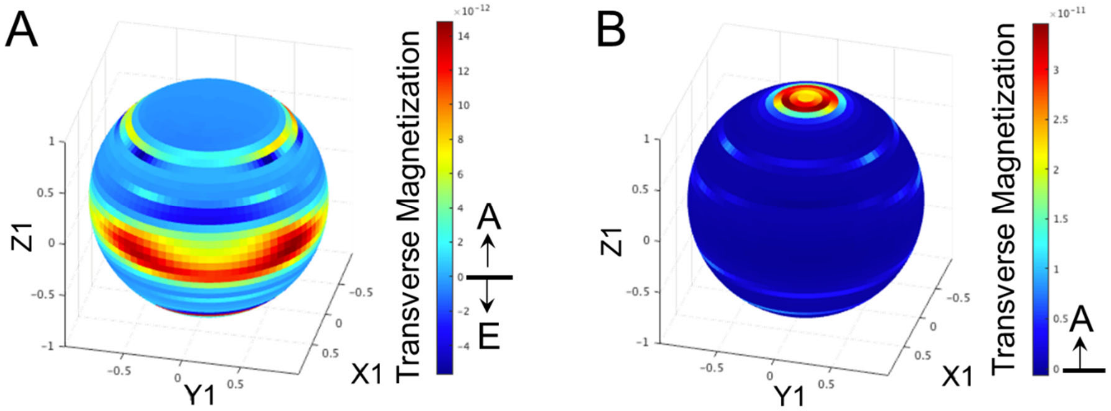

Supplementary Figure 21. (A) Mapping of the transverse magnetization at the field strength of "X, Y" in Supplementary Figure 22A from the spin polarized ESR spectrum of ^{5}TT state. (B) Mapping of the magnetizations for the field strength of "Z" in Supplementary Figure 22. The mappings of the magnetization were performed from the computations of electron spin polarization[1] for all possible directions of the external magnetic field as reported previously[2,3].

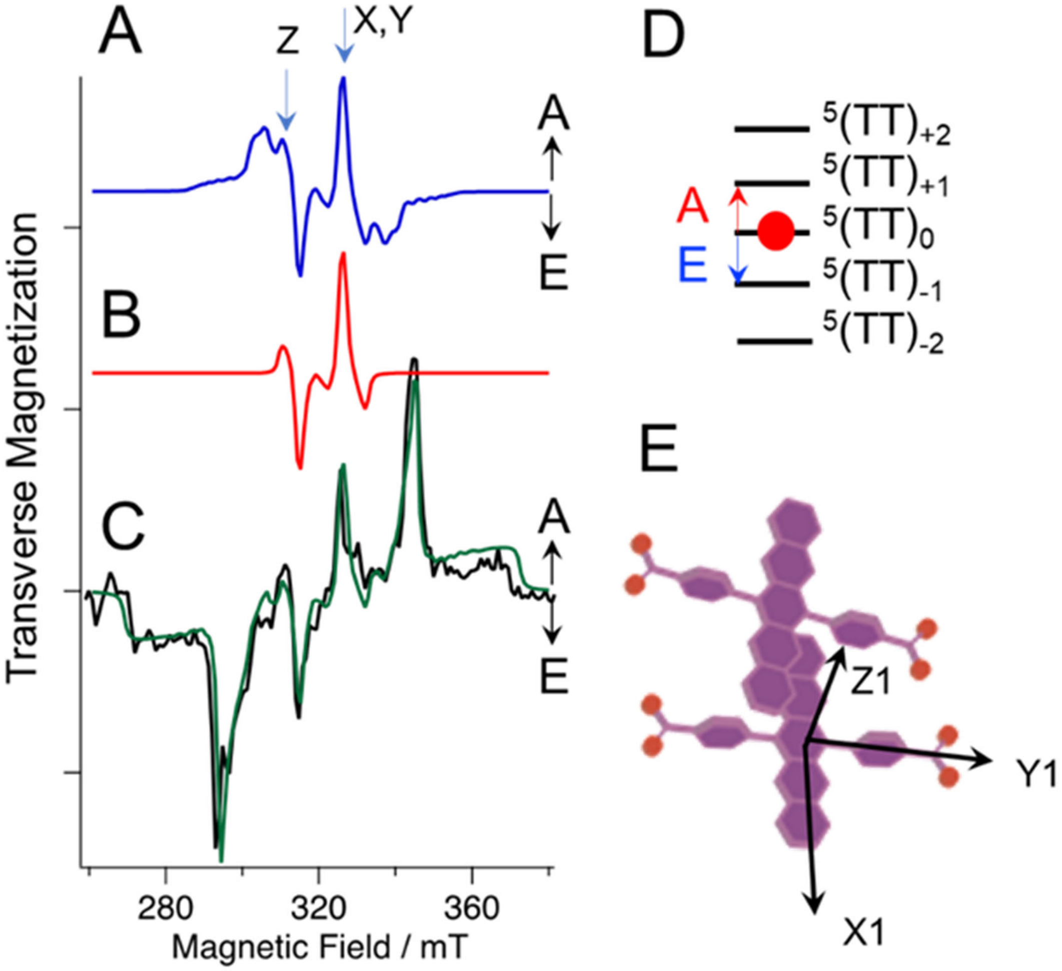

Supplementary Figure 22. (A) Computed ESR spectrum of ^{5}TT state obtained by the powder pattern calculation with considering the computed spin sublevel populations in $^5(TT)_{+2}$, $^5(TT)_{+1}$, $^5(TT)_0$, $^5(TT)_{-1}$ and $^5(TT)_{-2}$ in (D) both of TT_A and TT_B states, by using the reported method[1]. (B) Computed ESR spectrum of ^{5}TT state obtained by the powder pattern calculation only by $^5(TT)_0 \rightarrow {}^5(TT)_{+1}$ and by $^5(TT)_0 \rightarrow {}^5(TT)_{-1}$ contributions in (D). (C) Experimental ESR spectrum of NaPDBA (black line) and simulated ESR spectrum (green line) composed of the blue line in (A) and of the isolated triplet state generated by the ISC. (E) Conformations of the TT_A and TT_B states undergoing the mutual J-modulation between J_A = -5.0 T and J_B = -0.8 T in the TT_A and TT_B, respectively in the exchange-coupling (J). This model is used for the present calculations in the spin sublevel populations and the magnetizations.

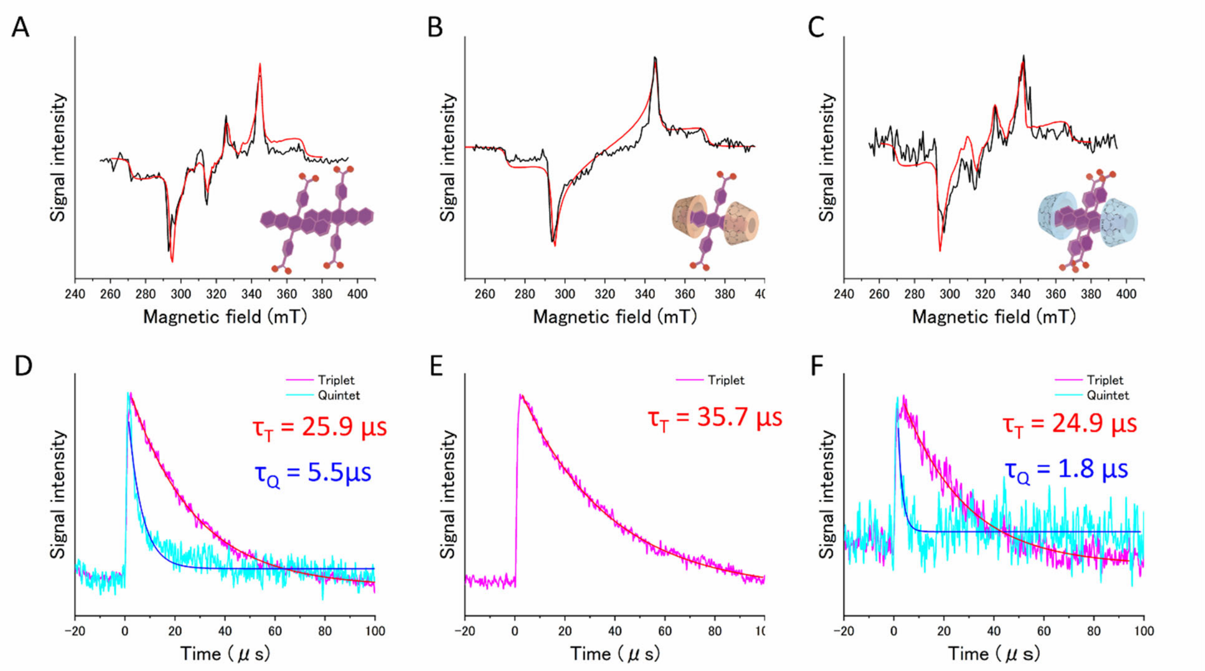

Supplementary Figure 23. Time-resolved ESR spectra (A-C) and decays (D-F) of (A, D) NaPDBA, (B, E) NaPDBA-βCD, and (C, F) NaPDBA-γCD in water-glycerol (1:1) at 143 K ([NaPDBA] = 1 mM, [βCD] = [γCD] = 5 mM) just after photoexcitation at 527 nm. Fitting parameters of simulated spectra (red lines in A-C) for ISC-born triplet and SF-born quintet are summarized in Supplementary Table1 and 2, respectively. The results of single exponential fitting is shown in D-E with corresponding decay times.

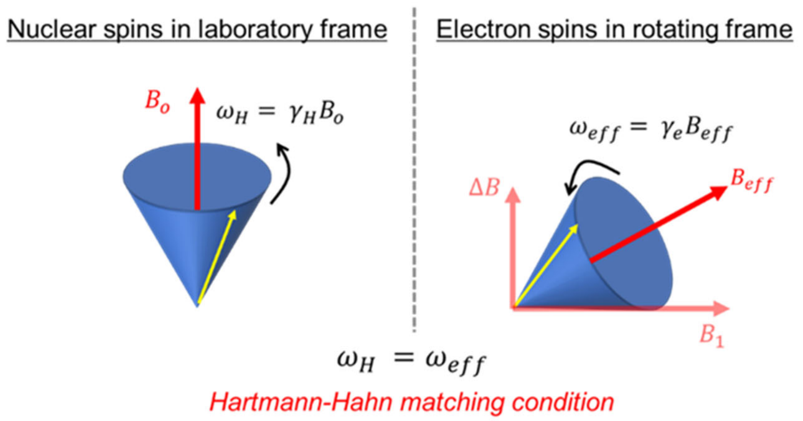

Supplementary Figure 24. Schematic illustration of the Hartmann-Hahn matching condition. The nuclear spins precess with the Larmor frequency ω_H around the external magnetic field B_o in the laboratory frame. The electron spins precess around the effective magnetic field $B_{eff} = \sqrt{{B_1}^2 + \Delta B^2}$ with the frequency $\omega_e = \gamma_e B_{eff}$ in the rotating frame, where B_1 is the power of the irradiated microwave perpendicular to the external magnetic field, ΔB is the offset between ω_{e0} and ω_{MW} $(\Delta B = (\omega_{e0} - \omega_{MW})/\gamma_e)$. When the Hartmann‑Hahn matching condition $\omega_H = \omega_{eff}$ is satisfied, the polarization transfer occurred most efficiently [4-7].

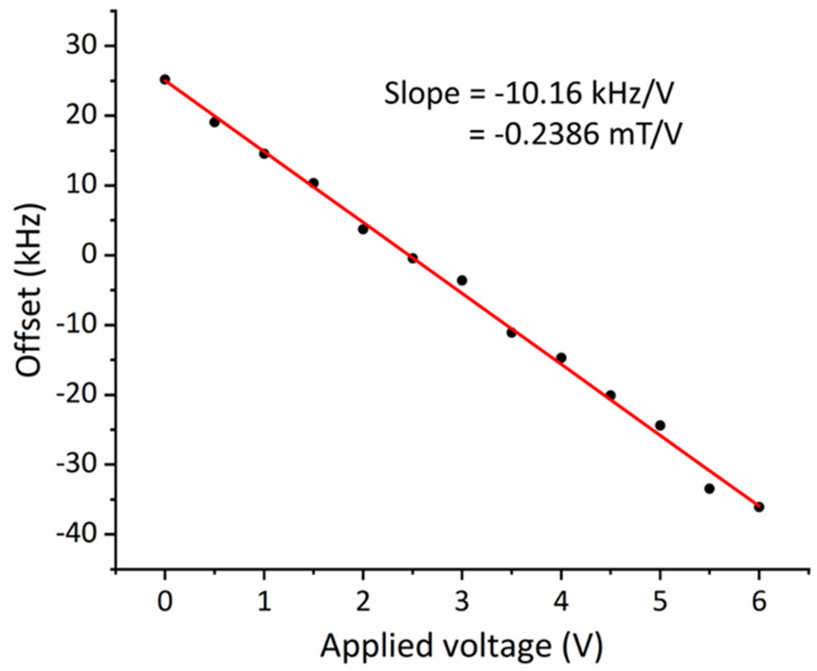

Supplementary Figure 25. Offsets from the NMR resonance frequency at 27.98 MHz when various voltages are applied to the field sweep circuit. Deionized water was used for NMR measurement. A 50 V sweep in the ISE sequence in this study corresponds to a 10 mT sweep.

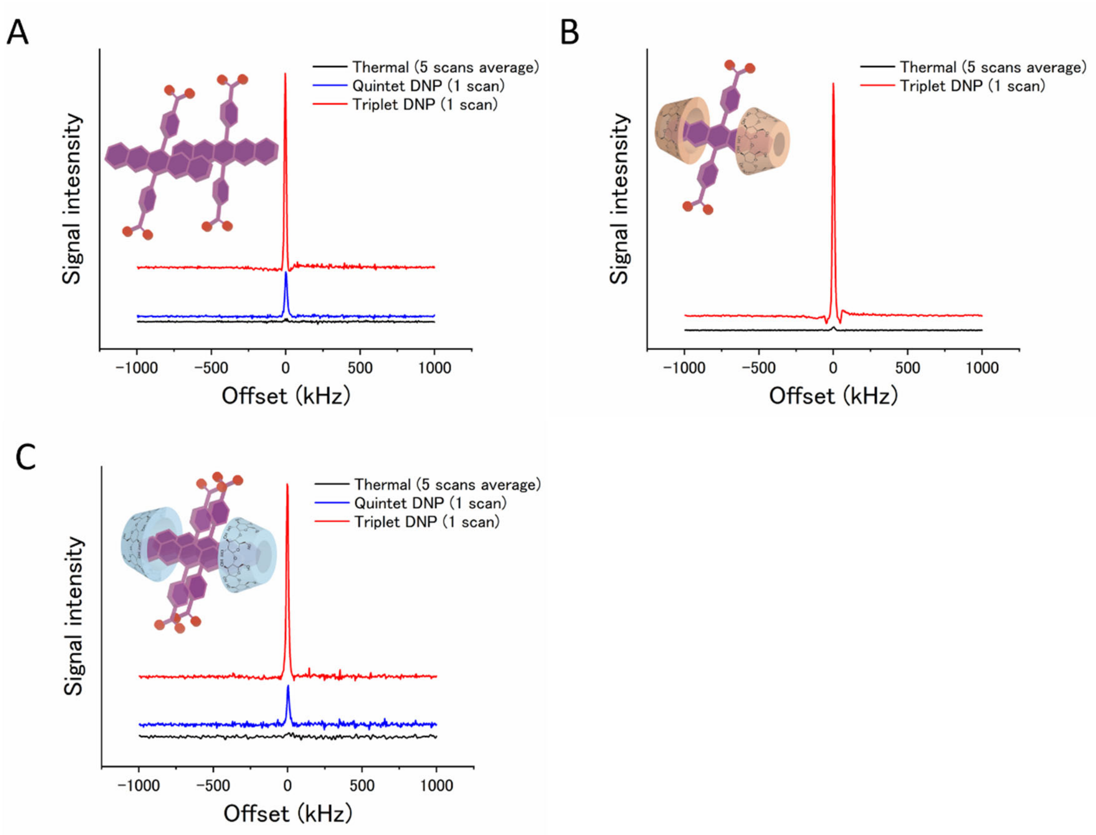

Supplementary Figure 26. ^{1}H-NMR signals of under thermal conditions (black lines, 5 scans every 10 min), after quintet-DNP (blue lines, ISE sequence for 5 min, 1 scan), and after triplet-DNP (red lines, ISE sequence for 5 min, 1 scan) of water-glycerol (glycerol-d_8:D_2O:H_2O = 5:4:1) containing (A) NaPDBA, (B) NaPDBA-βCD, and (C) NaPDBA-γCD at 100 K ([NaPDBA] = 1 mM, [βCD] = [γCD] = 5 mM). Photo-excitation wavelength and frequency were 527 nm and 500 Hz, respectively. DNP was performed by matching the magnetic field to the triplet (27.4 MHz (A,B), 27.3 MHz (C)) and quintet peaks(26.9 MHz (A, C)), respectively. Microwave power: 40 W (triplet), 20 W(quintet); laser power: 2.7 W (A, B), 1.5 W (C); magnetic field sweep width: 25 μs (triplet), 10 μs (quintet).

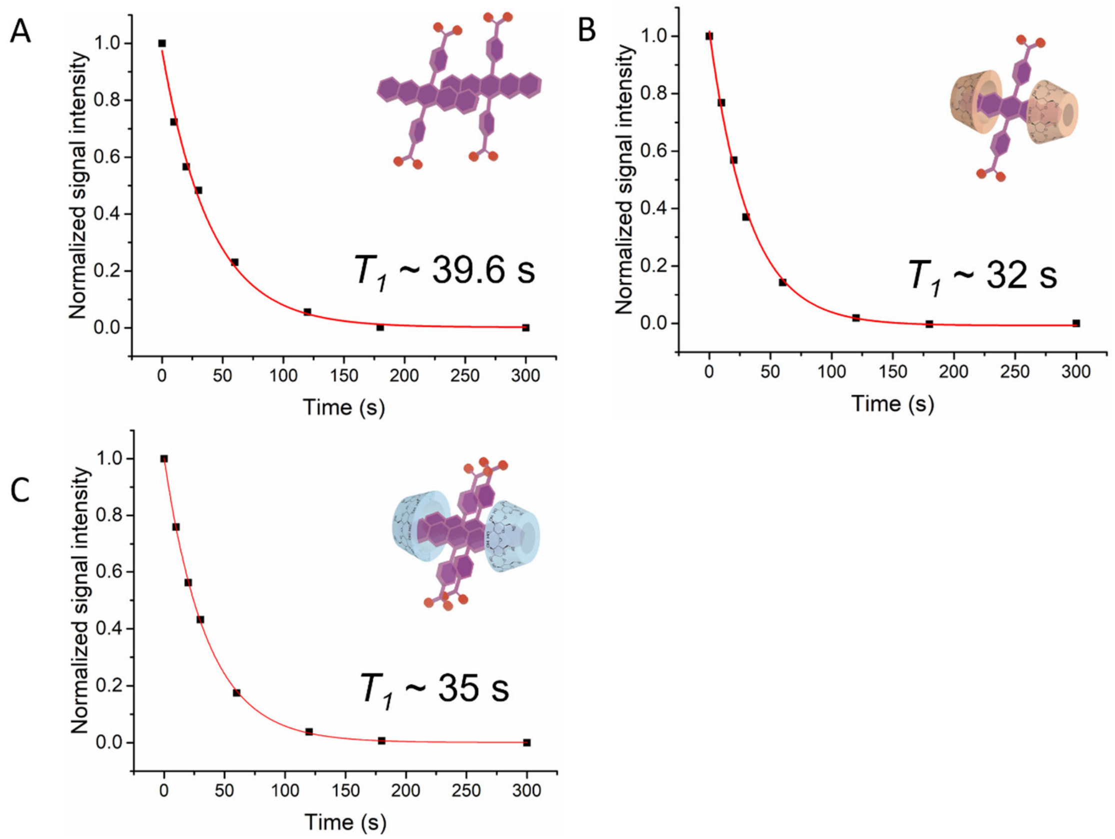

Supplementary Figure 27. Decay of ^{1}H NMR signal after triplet-DNP of water-glycerol (glycerol-d_8:D_2O:H_2O = 5:4:1) containing (A) NaPDBA, (B) NaPDBA-βCD, and (C) NaPDBA-γCD at 100 K ([NaPDBA] = 1 mM, [βCD] = [γCD] = 5 mM). Triplet-DNP was performed at 27.4 MHz (ISE sequence for 30 s and 1 scan; microwave power: 40 W; laser power: 1.5 W; magnetic field sweep width: 25 μs). The results of single-exponential fitting are shown as red lines.

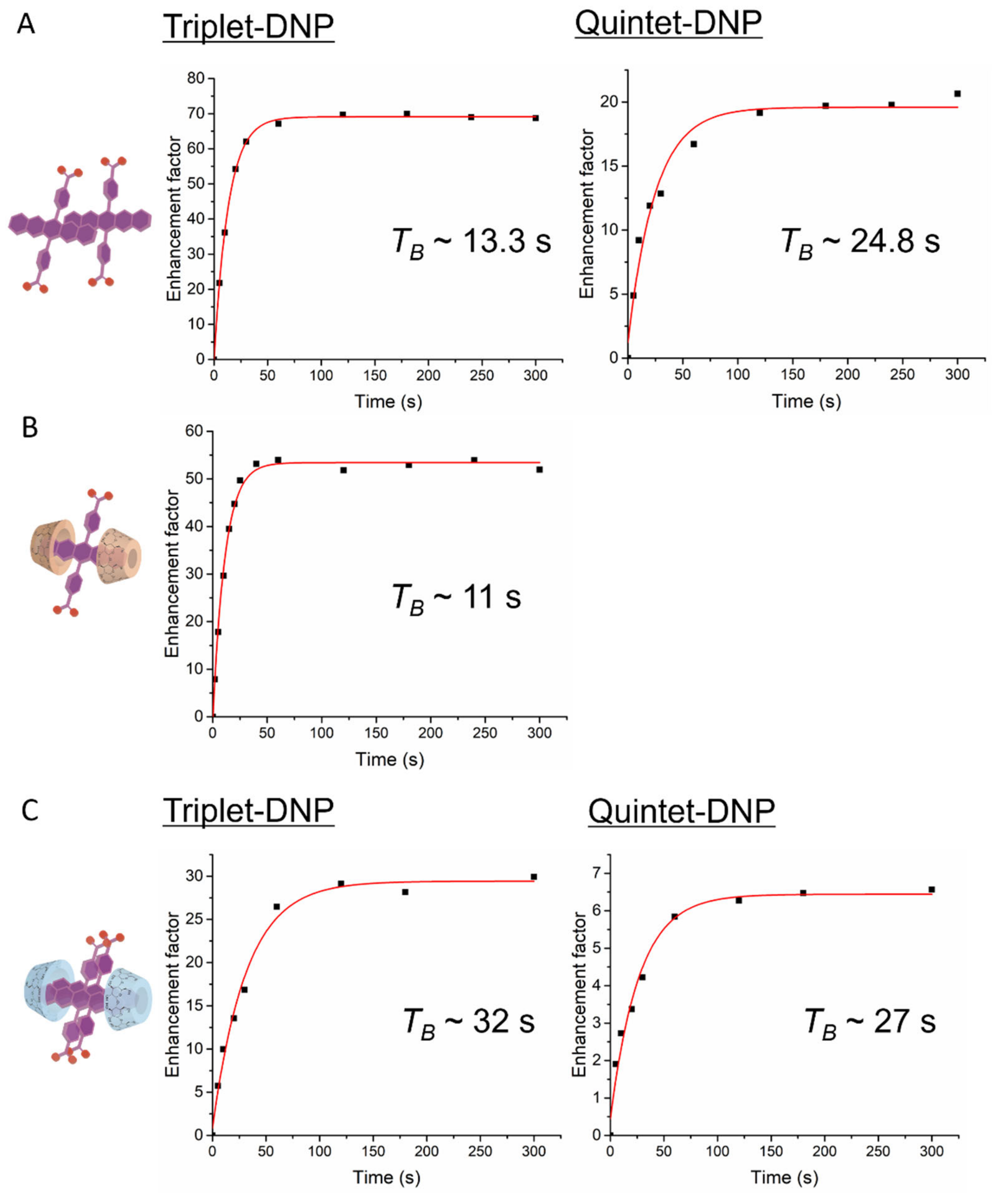

Supplementary Figure 28. Polarization build-up curves of triplet-DNP and quintet-DNP of water-glycerol (glycerol-d_8:D_2O:H_2O = 5:4:1) containing (A) NaPDBA, (B) NaPDBA-βCD, and (C) NaPDBA-γCD at 100 K ([NaPDBA] = 1 mM, [βCD] = [γCD] = 5 mM). Photo-excitation wavelength and frequency were 527 nm and 500 Hz, respectively. Triplet-DNP was performed at 27.4 MHz (A, B) and 27.3 MHz (C) (1 scan; microwave power: 40 W; laser power: 2.7 W (A, B), 1.5 W (C); magnetic field sweep width: 25 μs). Quintet-DNP was performed at 26.9 MHz (1 scan; microwave power: 20 W; laser power: 2.7 W (A, B), 1.5 W(C); magnetic field sweep width: 10 μs). The results of single-exponential fittings are shown as red lines.

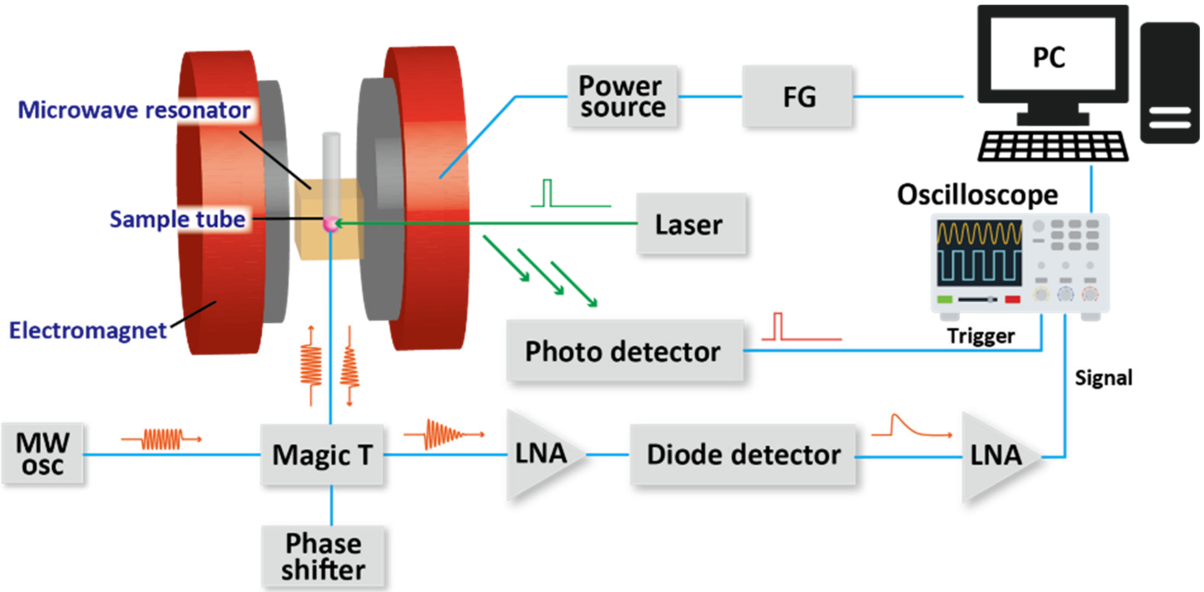

Supplementary Figure 29. Setup for time-resolved ESR. LNA: low noise amplifier; MW osc: microwave oscillator; FG: function generator. Adopted with permission from Yanai, Nobuhiro. Triplet Dynamic Nuclear Polarization of Guest Molecules through Induced Fit in a Flexible Metal–Organic Framework. 2021. Wiley Online Library, 10.1002/anie.202115792.

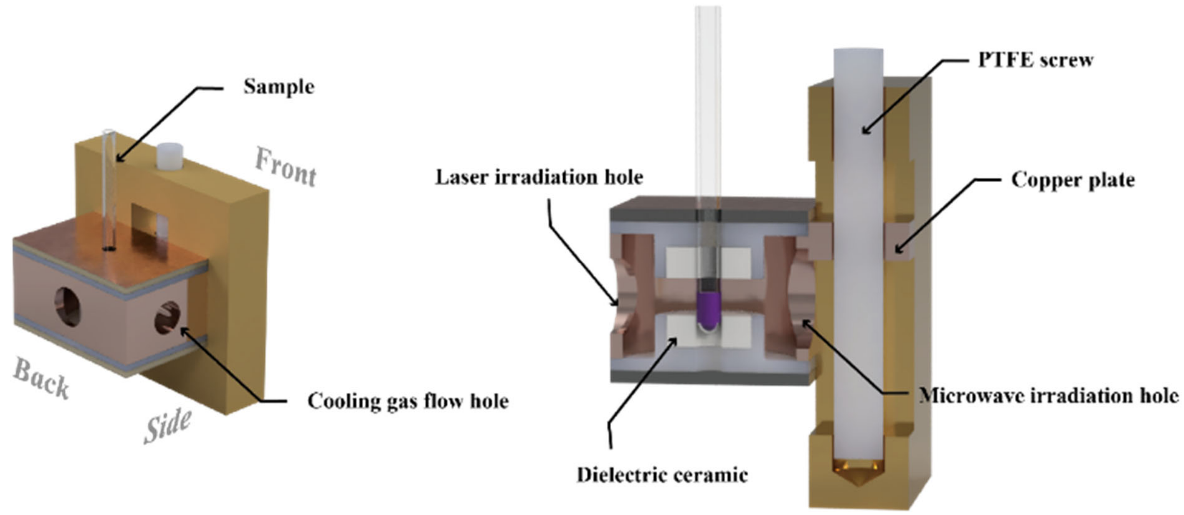

Supplementary Figure 30. The cavity resonator in the time-resolved ESR setup. Overall view (left) and cross-sectional view (right). Adopted with permission from Yanai, Nobuhiro. Triplet Dynamic Nuclear Polarization of Guest Molecules through Induced Fit in a Flexible Metal–Organic Framework. 2021. Wiley Online Library, 10.1002/anie.202115792.

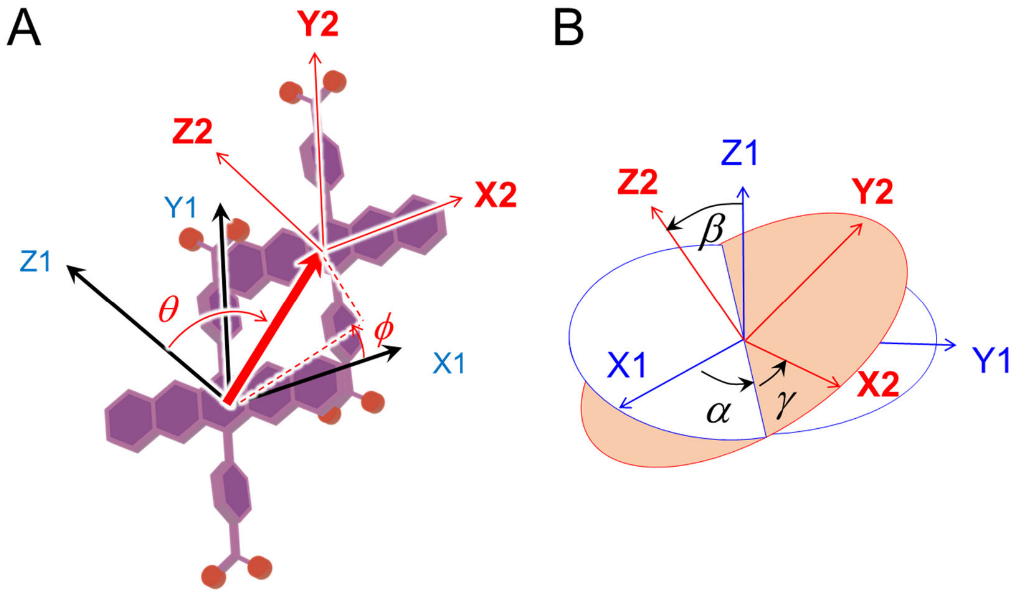

Supplementary Figure 31. Angles defined for geometry of the triplet pair for the ZFS principal axes represented by X1, Y1, Z1 for triplet 1 and by X2, Y2, Z2 for triplet 2 in the TT multiexciton. (A) Polar angles, (θ, φ) and (B) Euler angles, (α, β, γ).

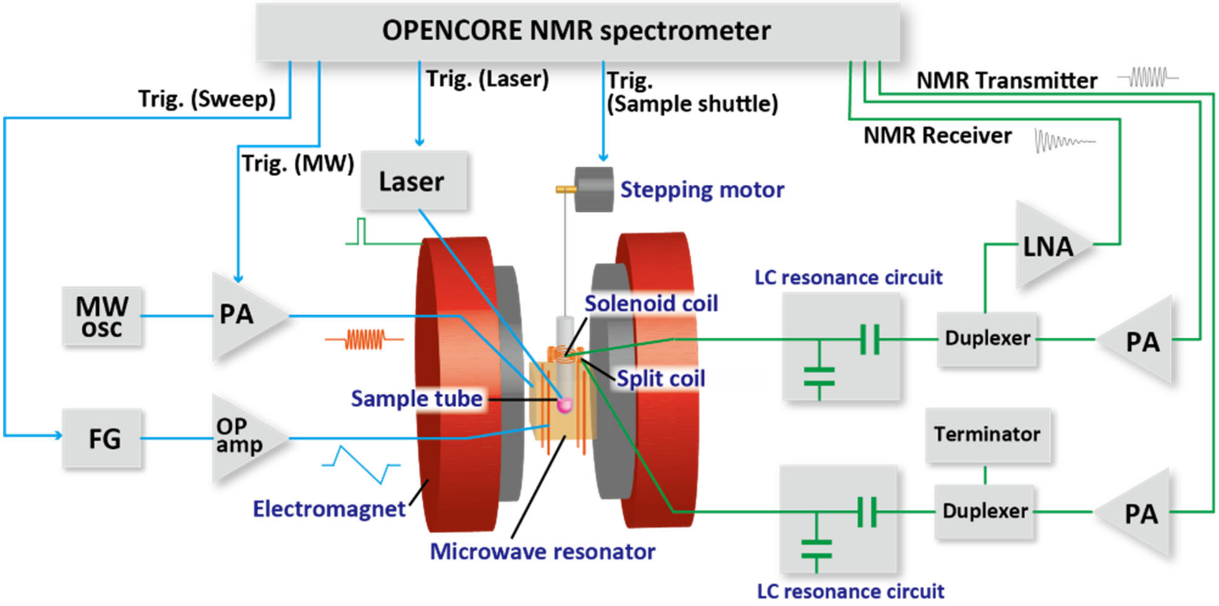

Supplementary Figure 32. Setup for triplet-DNP. PA: power amplifier; MW osc: microwave oscillator; FG: function generator; Trig.: TTL trigger signal; LNA: low noise amplifier. Adopted with permission from Yanai, Nobuhiro. Triplet Dynamic Nuclear Polarization of Guest Molecules through Induced Fit in a Flexible Metal–Organic Framework. 2021. Wiley Online Library, 10.1002/anie.202115792.

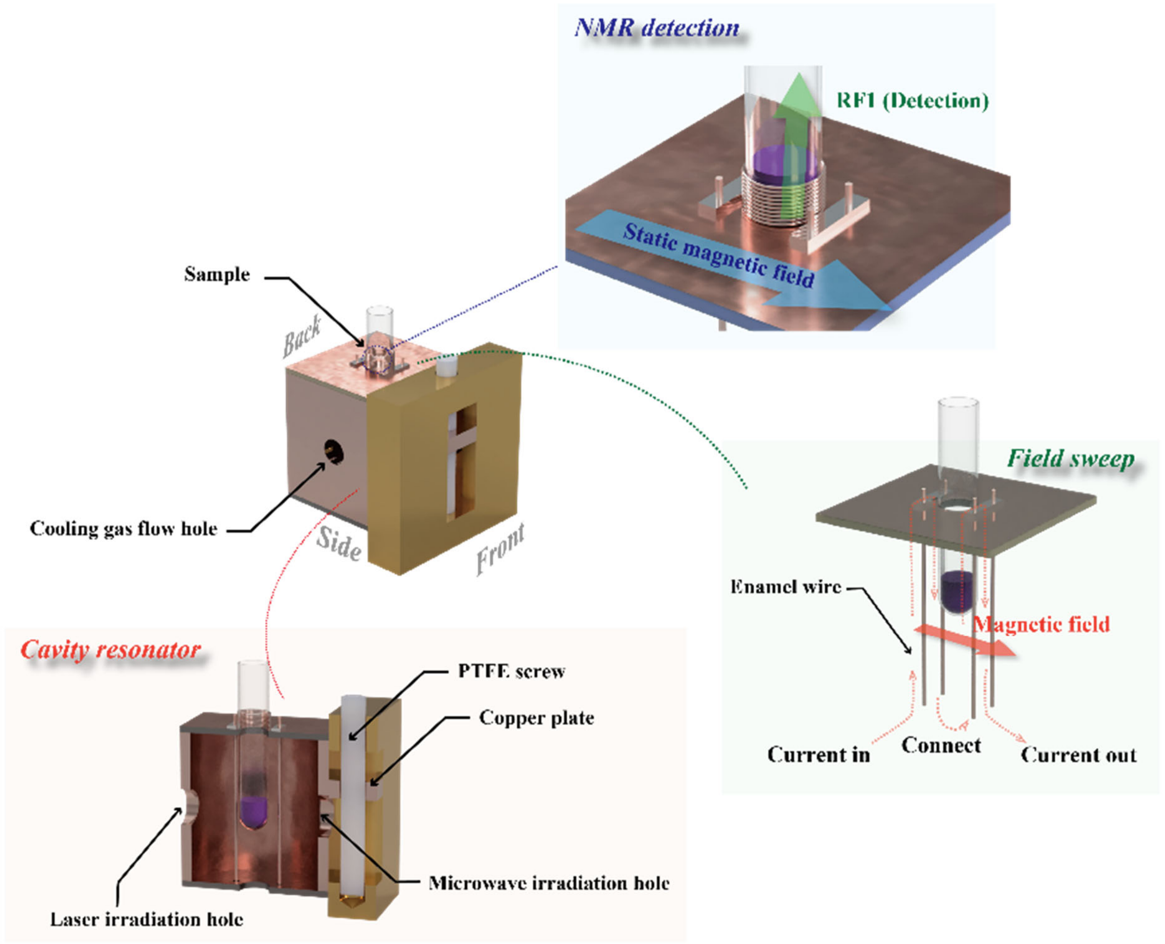

Supplementary Figure 33. The resonator used in triplet-DNP. NMR detection part (blue), field sweep part (green) and cavity resonator (red). Adopted with permission from Yanai, Nobuhiro. Triplet Dynamic Nuclear Polarization of Guest Molecules through Induced Fit in a Flexible Metal–Organic Framework. 2021. Wiley Online Library, 10.1002/anie.202115792.

Supplementary Table 1. Fitting parameters used for ISC-born triplet in the time-resolved ESR spectra (Fig. 4 and S23).

State	Relative populations	*D* /MHz	*E* /MHz
NaPDBA	T_x = 0.09<br>T_y = 0.91<br>T_z = 0	1380	-20
NaPDBA-βCD	T_x = 0.09<br>T_y = 0.91<br>T_z = 0	1410	-15
NaPDBA-γCD	T_x = 0.09<br>T_y = 0.91<br>T_z = 0	1380	-15

Supplementary Table 2. Fitting parameters used for SF-born quintet in the time-resolved ESR spectra (Fig. 4 and S23).

<table>
<tr><th colspan="2">State</th><th>Populations a)</th><th>J /GHz</th><th>D /MHz</th><th>E /MHz</th><th>D_{SS} /MHz</th><th>Euler angles b) /degrees</th><th>Polar angles b) /degrees</th><th>$\tilde{\upsilon}_{vib}$ /cm^{-1}</th><th>k_{REC} /s^{-1} c)</th></tr>
<tr><td rowspan="2">NaPDBA</td><td>TT_1</td><td rowspan="2">$Q_{+2} = 0.118$
$Q_{+1} = 0.024$
$Q_0 = 0.099$
$Q_{-1} = 0.027$
$Q_{-2} = 0.133$</td><td>-40</td><td rowspan="2">1180</td><td rowspan="2">-20</td><td rowspan="2">-120</td><td>$\alpha = 0$
$\beta = 0$
$\gamma = 0$</td><td rowspan="2">$\theta = 35$
$\varphi = 0$</td><td rowspan="2">21.5</td><td>1.0×10^6</td></tr>
<tr><td>TT_2</td><td>-8</td><td>$\alpha = 90$
$\beta = 20$
$\gamma = -90$</td><td>-</td></tr>
<tr><td rowspan="2">NaPDBA-γCD</td><td>TT_1</td><td rowspan="2">$Q_{+2} = 0.087$
$Q_{+1} = 0.008$
$Q_0 = 0.074$
$Q_{-1} = 0.009$
$Q_{-2} = 0.103$</td><td>-60</td><td rowspan="2">1180</td><td rowspan="2">-20</td><td rowspan="2">-150</td><td>$\alpha = 0$
$\beta = 0$
$\gamma = 0$</td><td rowspan="2">$\theta = 35$
$\varphi = 0$</td><td rowspan="2">21.5</td><td>2.0×10^6</td></tr>
<tr><td>TT_2</td><td>-20</td><td>$\alpha = 90$
$\beta = 10$
$\gamma = -90$</td><td>-</td></tr>
</table>

a) These values were obtained as ensemble averaged sublevel populations for the field orientations perpendicular to the Z principal axis of the zero-field splitting interaction of the triplet molecule in the multiexciton at $t = 0.4$ μs. This procedure was undertaken because the $^5(TT)_0 \rightarrow {}^5(TT)_1$ microwave transition in Fig. 4C-D was utilized for the DNP experiments and is dominantly contributed by these field orientations to satisfy the microwave resonance at the field strength. The residual populations were obtained in the excited complexes, as follows: $S_0S_0 = 0.541$, $^1(TT) = 0.057$ for NaPDBA and $S_0S_0 = 0.646$, $^1(TT) = 0.074$ for NaPDBA-γCD. To fit the entire ESR spectra, however, computed anisotropic spin polarization patterns were averaged for all the possible field orientations to obtain the powder-pattern spectra, as reported previously [1]. Fitting parameters are also detailed in this reference.

b) The polar angles correspond to θ and φ in Supplementary Figure 31A, and the Euler angles correspond to α, β and γ in Supplementary Figure 31B.

c) The singlet recombination constant k_{REC} represents the rate of singlet TT dimer $^1(TT)$ deactivation to the ground state. $^1(TT)$ and $^5(TT)$ are assumed to be in equilibrium. The k_{REC} value was estimated based on change in ESR signal intensity from immediately after photoexcitaton to 2-3 μs later. The change of ESR signal intensity was well explained with k_{REC} of 1.0×10^6 s^{-1} for NaPDBA and 2.0×10^6 s^{-1} for NaPDBA-γCD.

Supplementary Table 3. Matrix elements of the magnitudes (10^{11} rad/s) of the spin Hamiltonian of [abs($\mathbf{H}_{TTB}$)] calculated for the TT_B state with the B_0 direction of (θ_B, ϕ = (90°, 45°)) of the external magnetic field in the (X1, Y1, Z1) coordinate in Figure R1 in the presence of the exchange coupling of J_B = -0.8 T.

	$^5TT_{+2}$	$^5TT_{+1}$	$^5TT_0$	$^5TT_{-1}$	$^5TT_{-2}$	$^3TT_{+1}$	$^3TT_0$	$^3TT_{-1}$	^1TT
$^5TT_{+2}$	3.9231	0.0004	0.0001	0.0000	0.0000	0.0010	0.0007	0.0000	0.0345
$^5TT_{+1}$	0.0004	3.3931	0.0002	0.0001	0.0000	0.0016	0.0007	0.0005	0.0019
$^5TT_0$	0.0001	0.0002	2.8395	0.0002	0.0001	0.0012	0.0000	0.0012	0.0343
$^5TT_{-1}$	0.0000	0.0001	0.0002	2.2637	0.0004	0.0005	0.0007	0.0015	0.0018
$^5TT_{-2}$	0.0000	0.0000	0.0001	0.0004	1.6676	0.0000	0.0007	0.0010	0.0379
$^3TT_{+1}$	0.0010	0.0016	0.0012	0.0005	0.0000	2.2371	0.0003	0.0001	0.0000
$^3TT_0$	0.0007	0.0007	0.0000	0.0007	0.0007	0.0003	2.8485	0.0003	0.0000
$^3TT_{-1}$	0.0000	0.0005	0.0012	0.0015	0.0010	0.0001	0.0003	3.3665	0.0000
^1TT	0.0345	0.0019	0.0343	0.0018	0.0379	0.0000	0.0000	0.0000	5.6349

While the interaction between the ^5TT and ^1TT is large for generating $^5TT_{\pm 2}$ and $^5TT_0$ with the coupling values of 3.5×10^9 rad/s, the interactions between ^3TT and ^1TT is zero. Also, the interactions between ^5TT and ^3TT minor being ca. 10^8 rad/s. Thus, the generations of the ^3TT states are neglected in the present multiexciton. It is thus concluded that the ^3TT is not created from the quintet multiexciton in the presence of the strong exchange coupling.

Supplementary Table 4. Number of molecules used for each MD simulations.

System	Number of molecules			
	NaPDBA	β/γCD	water	glycerol
NaPDBA in water	20	0	167054	0
NaPDBA in water-glycerol	20	0	74487	18500
NaPDBA-βCD in water-glycerol	4	20	97423	24300
NaPDBA-γCD in water-glycerol	4	20	97055	24300
NaPDBA-βCD in water	4	20	215005	0
NaPDBA-γCD in water	4	20	214845	0

Supplementary References

1 Kobori, Y., Fuki, M., Nakamura, S. & Hasobe, T. Geometries and Terahertz Motions Driving Quintet Multiexcitons and Ultimate Triplet-Triplet Dissociations via the Intramolecular Singlet Fissions. *J. Phys. Chem. B* **124**, 9411-9419, doi:10.1021/acs.jpcb.0c07984 (2020).

2 Hasegawa, M. *et al.* Regulated Electron Tunneling of Photoinduced Primary Charge-Separated State in the Photosystem II Reaction Center. *J. Phys. Chem. Lett.* **8**, 1179-1184, doi:10.1021/acs.jpclett.7b00044 (2017).

3 Kobori, Y., Ako, T., Oyama, S., Tachikawa, T. & Marumoto, K. Transient Electron Spin Polarization Imaging of Heterogeneous Charge-Separation Geometries at Bulk-Heterojunction Interfaces in Organic Solar Cells. *J. Phys. Chem. C* **123**, 13472-13481, doi:10.1021/acs.jpcc.9b02672 (2019).

4 Henstra, A., Ducksen, P., Schmidt, J. & Wenckebach, W. T. Nuclear Spin Orientation via Electron Spin Locking (NOVEL). *J. Magn. Reson.* **77**, 389-393 (1988).

5 Henstra, A., Schmidt, J., Lin, T.-S. & Wenckebach, W. T. High dynamic nuclear polarization at room temperature. *Chem. Phys. Lett.* **165**, 6-10 (1990).

6 Henstra, A. & Wenckebach, W. T. The theory of nuclear orientation via electron spin locking (NOVEL). *Mol. Phys.* **106**, 859-871, doi:10.1080/00268970801998262 (2008).
7 Nishimura, K. *et al.* Materials chemistry of triplet dynamic nuclear polarization. *Chem. Commun.* **56**, 7217-7232, doi:10.1039/d0cc02258f (2020).
8 Fujiwara, S., Matsumoto, N., Nishimura, K., Kimizuka, N., Tateishi, K., Uesaka, T., & Yanai, N. Triplet Dynamic Nuclear Polarization of Guest Molecules through Induced Fit in a Flexible Metal–Organic Framework. *Angew. Chem. Int. Ed.*, **61**, e202115792 (2022).
